# Kat6a functions to protect lower jaw patterning during cellular stress

**DOI:** 10.21203/rs.3.rs-10009504/v1

**Published:** 2026-10-06

**Authors:** Ritika Ghosal, Mary E Swartz, Joanna Liu, Anushree Patel, Johann K Eberhart

**Affiliations:** Department of Molecular Biosciences and Waggoner Center for Alcohol and Addiction Research, University of Texas at Austin, USA

## Abstract

Phenotypic outcomes of most birth defects involve complex multifactorial interactions. In humans, heterozygosity for *KAT6A* or *MEF2C* causes highly variable syndromes^1,2^. These syndromes overlap phenotypically with Fetal Alcohol Syndrome (FAS)^2,3,4^, with defects to the pharyngeal arches-derived craniofacial skeleton. In zebrafish, we demonstrate that cellular stressors, like ethanol, induce ventral patterning defects specifically in the first pharyngeal arch in *kat6a* mutants and heterozygotes and result in lower jaw defects. This is striking as anteroposterior and dorsoventral patterning of pharyngeal arches are typically independent. Kat6a regulates *hox* gene expression for anteroposterior patterning. Bmp/Edn1 signaling and their targets, like *dlx* genes and *hand2*, regulate dorsoventral patterning. In exposed *kat6a* mutants and heterozygotes, Bmp/Edn1 signaling remains intact. However, exposure causes a first arch specific loss of ventral specifiers in mutants and heterozygotes. The overall phenotype resembles loss of *mef2ca*, which governs dorsoventral patterning independent of Bmp/Edn1. We find that *mef2ca* is selectively lost in the first pharyngeal arch of exposed *kat6a* mutants and heterozygotes. CRISPR knockdown of *mef2ca* phenocopies the effect of exposure in *kat6a* mutants and heterozygotes. Tissue mosaics confirmed that first arch neural crest specifically require *kat6a*. Thus, multifactorial interactions reveal an unknown link between pharyngeal arch anteroposterior and dorsoventral patterning.

Development of the vertebrate face requires complex signaling interactions between neural crest-derived pharyngeal mesenchyme and the pharyngeal epithelia. Neural crest cells (NCCs) migrate from the dorsal neural tube to specific pharyngeal arches carrying with them intrinsic anteroposterior identities of the skeletal elements formed by their destination arch^5,6^. Conversely, local signaling from the pharyngeal epithelia pattern NCCs along the dorsoventral axis within the arches^7,8^. The prevailing view holds that anteroposterior and dorsoventral patterning of pharyngeal arch series are established independent of one another through separate sets of signaling pathways. For instance, loss of *hox2* function causes the homeotic transformation of the second (hyoid) arch to the first (mandibular) arch^9,10^. Despite losing its anteroposterior identity, the transformed hyoid arch undergoes normal dorsoventral patterning to form duplicate upper and lower jaw skeleton. Similarly, loss of Bmp (Bone Morphogenetic Protein) and Edn1 (Endothelin1) signaling disrupts dorsoventral patterning affecting ventral and intermediate arch specification without altering their anteroposterior identity^11,12^. Because anteroposterior and dorsoventral patterning require distinct signaling pathways, they are considered to be independent of one another.

Kat6a (Lysine acetyltransferase 6a) is a chromatin modifier that plays a key role in craniofacial development^13^. A known function of Kat6a is anteroposterior patterning of hyoid and posterior arches via maintenance of *hox* expression. Similar to *hox2*-deficient embryos, *kat6a* mutants lose hyoid arch identity, resulting in a duplicated set of the mandibular arch skeleton^14^. In humans, heterozygosity for *KAT6A* causes KAT6A syndrome with variable craniofacial and cognitive defects^2,15^. The range of severity in KAT6A syndrome cannot be fully explained by *KAT6A* genotype alone. Studies in mice show that phenotypic severity of loss of *Kat6a* was dependent on genetic background and double heterozygosity for *Tbx1*and *Kat6a* results in cardiac defects^13,16^. In addition to genetic background, environmental factors can contribute substantially to the phenotypic outcome of a mutation resulting in clinical heterogeneity, as is seen in holoprosencephaly^17,18^.

We found that embryonic exposures to common teratogens cause a dorsoventral patterning defect that is isolated to the first pharyngeal arch in zebrafish *kat6a* mutants and heterozygotes. Unexposed *kat6a* mutants develop largely normal mandibular arch derivatives. Exposed mutants frequently have loss of the lower jaw (Meckel’s cartilage) and fusion of the Meckel’s cartilage and upper jaw (palatoquadrate). While unexposed heterozygotes develop normally, exposure induced similar craniofacial defects in *kat6a* heterozygotes, thereby mimicking the dominant effect observed in humans. Disruption of signaling centers in the anterior foregut endoderm or oral ectoderm can cause first arch specific defects^28, 29^. However, markers of the pharyngeal endoderm and oral ectoderm (*shha, fgf8a* and *pitx2*) were unaffected in ethanol-exposed mutants and heterozygotes. Components of Bmp and Endothelin1 signaling pathways were also normally expressed. Interestingly, a subset of ventral and intermediate arch specifier genes, such as *hand2*, *dlx5a*, and *dlx4b*, were specifically lost in the mandibular, but not hyoid arch, of ethanol-exposed *kat6a* mutants and, to a lesser extent, heterozygotes. This same set of specifier genes are lost in *mef2ca* mutants, although with *mef2ca* the effect is not first arch specific. We find a first arch specific loss of *mef2ca* in exposed *kat6a* mutants and heterozygotes. Knockdown of *mef2ca* via CRISPR/Cas9 phenocopies the effect of exposure in *kat6a* mutants and heterozygotes. Using tissue mosaics, we demonstrate that *kat6a* is required autonomously in first arch neural crest mesenchyme.

In summary, we reveal a previously unknown developmental link between anteroposterior and dorsoventral patterning during pharyngeal arch morphogenesis. Loss of *KAT6A* or *MEF2C* causes dominant human diseases and the exposures that we examine are common. There is phenotypic overlap in KAT6A syndrome, MEF2C haploinsufficiency syndrome and FAS, which includes defects to the lower jaw^2,3,4^. We uncover a mechanistic association that helps explain the phenotypic similarity between FAS and these two genetic syndromes. The involvement of environmental exposures has been unexplored in KAT6A syndrome and MEF2C haploinsufficiency syndrome. However, there is phenotypic variability within families with inherited KAT6A mutations suggesting an environmental component^19^. Thus, our findings shed new light on human disease and phenotypic plasticity.

## Kat6a buffers against cell stressors

Zebrafish *kat6a* mutants lose anteroposterior identity in second and more posterior pharyngeal arches due to loss of *hox1-4* expression^14^. Consequently, the hyoid arch undergoes a homeotic transformation to form a second set of mandibular arch-like skeletal elements. This transforms the ceratohyal cartilages to mirror-image duplicated Meckel’s cartilages with their posterior attachment on the jaw joint^14^. Hox-negative mandibular arch develops normally to form Meckel’s cartilage, palatoquadrate and anterior neurocranium in *kat6a* mutants^14^. However, we found that exposure to common teratogens such as ethanol, nicotine, and piperonyl butoxide (PBO, a pesticide synergist commonly found in indoor air) at sub-teratogenic doses induced defects in Meckel’s cartilage in *kat6a* mutants (Fig. 1; Extended Data Fig. 1). Even the carrier dimethyl sulfoxide (DMSO), which can have teratogenic effects^20^, elicited lower jaw defects in a small number of mutants (Extended Data Fig. 1c). These results collectively suggest an unknown *hox*-independent role of *kat6a* in the mandibular arch. This role buffers against the deleterious effects of environmental exposures that are very common in human and, thus, has critical implications relating to human birth defects. To understand how environmental stressors can modulate *kat6a* mutant phenotypes, we focused on ethanol since an estimated 1.1-5% of births in the US are affected by Fetal Alcohol Spectrum Disorder (FASD)^21,22^.

Wildtype zebrafish embryos exposed to 1% ethanol from gastrulation (6 hpf, hours post fertilization) to first feeding larval stage (4 dpf, day post fertilization) do not have gross craniofacial skeletal defects^23,24^. This dose of ethanol results in a tissue level ethanol concentration equivalent to a ~.19-.28 blood alcohol concentration. Ethanol induced severe jaw defects in *kat6a* mutants (Fig. 1a), ranging from partial to complete loss of Meckel’s cartilage (Fig. 1b). Additionally, *kat6a* heterozygotes, that develop normally under control conditions, have craniofacial defects when exposed to ethanol (Fig. 1a). Ethanol does not alter *kat6a* expression so this effect must be mediated through other transcriptional targets of Kat6a (Extended Data Fig. 2). Ethanol also occasionally caused the loss of the jaw joint, resulting in the fusion of the Meckel’s cartilage and palatoquadrate (Fig. 1c). The transformed hyoid arch skeleton in ethanol-exposed *kat6a* mutants displayed no additional morphological defects or loss, with cartilage and joints being present in the second arch (Extended Data Fig. 3a). The neurocranium was intact in ethanol-exposed mutants (Extended Data Fig. 3a). A dose response demonstrated that mutants are sensitive to doses as low as 0.25%, equivalent to a 0.04725-0.069 blood alcohol concentration, although defects are most prevalent with 1% (Extended Data Fig. 4). We find that the shortest critical time window of sensitivity is between 10 hpf, the end of gastrulation, to 24 hpf, when neural crest cells have largely populated the pharyngeal arches (Fig. 1d). Unless otherwise noted, we use this 1% exposure from 10 hpf to 24 hpf for our analyses. This indicates that early development of the ventral mandibular arch is particularly sensitized to stressor-induced defects in *kat6a* mutants and heterozygotes.

We used linear measurements to quantify reductions in the size of craniofacial skeletal elements. In mutants and heterozygotes that generated Meckel’s cartilage, it was significantly reduced in size. Consistent with a first arch specific defect, the ceratohyal was not further reduced by ethanol in mutants. Strikingly, ethanol-exposed heterozygotes, but not mutants, had a significant reduction in anterior neurocranial elements and the ceratohyals (Extended Data Fig. 3b). Ethanol did not affect the mesodermal-derived posterior neurocranium^25^. This indicates that *kat6a*-ethanol interaction specifically affects the neural crest derivatives of the craniofacial skeleton.

We find that phenotypes are variable in ethanol exposed mutants, with the most common defects to the Meckel’s cartilage. Morphological characterization revealed that 76.92% (20/26) of the exposed mutants exhibited extremely reduced, hypoplastic Meckel’s cartilage. 53.84% (14/26) had broken or no Meckel’s cartilage, 61.53% (16/26) had reduced palatoquadrate or pterygoid process and 23.07% (6/26) had slight shape defects in anterior neurocranium (Extended Data Fig. 5d). Ethanol also induced fusions of cartilaginous elements in *kat6a* mutants with 30.76% (8/26) displaying fused Meckel’s cartilage and palatoquadrate (Extended Data Fig. 5b,d), and 11.53% (3/26) with fusion of ectopic cartilages to the anterior neurocranium (Extended Data Fig. 5c,d).

Phenotypes in heterozygotes were generally less penetrant. 19.35% (6/31) of heterozygotes exhibited breaks in Meckel’s cartilage (Extended Data Fig. 5a,e), 16.12% (5/31) displayed ventral hyoid skeletal defects (Extended Data Fig. 3a,5e), while 3.22% (1/31) exhibited slightly altered neurocranium shape (Extended Data Fig. 3a,5e). Ethanol exposed heterozygotes also exhibited frequent membrane bone defects, with opercle defects in 19.35% (6/31) and loss of branchiostegal ray in 45.16% (14/31) exposed heterozygotes (Extended Data Fig. 5e,f). Collectively, these data illustrate the preponderance of ethanol-induced defects within ventral mandibular arch derivatives in the mutants.

## Effect on mandibular crest mesenchyme

Neural crest specification and migration occur from 10-24 hpf^26^. However, we found no gross defects in neural crest migration into the arches at 24 hpf (Extended Data Fig. 6a). We also found no change in the number of proliferating neural crest cells (Extended Data Fig. 6b). We did find a significant increase in neural crest cell apoptosis in the mandibular arch of ethanol-exposed mutants, but not heterozygotes (Extended Data Fig. 7). Apoptosis may contribute to the reduction in the length of the cartilaginous elements observed in ethanol-exposed *kat6a* mutants. However, this cannot fully explain the specific loss of Meckel’s cartilage.

Studies in various vertebrate models have demonstrated the requirement of anterior foregut endoderm and surface ectoderm for craniofacial skeletal patterning^12,27,28,29^. The floor of the mouth in *kat6a* mutants is located more posterior than its position in the wildtype fish (Fig. 2a). This is likely due to the slightly more posterior location of the Meckel’s cartilage in *kat6a* mutants. In ethanol-exposed mutants, the oral cavity is more malformed with mis-patterned oral epithelium along the midline (arrowhead in Fig. 2a). However, ethanol does not affect *pitx2* expression in the oral ectoderm of *kat6a* mutants (Fig. 2b) indicating no intrinsic defect in the oral epithelium. We also found no alteration in *shha* or *fgf8a* expression (Fig. 2c), further indicating normal signaling from endoderm and oral ectoderm.

## Cell stress causes dorsoventral patterning defects in the mandibular arch of *kat6a* mutants and heterozygotes

We next examined the aboral ectoderm that regulates dorsoventral patterning through Bmp and Edn1 signaling. We found normal *bmp4* expression in the ventral ectoderm of ethanol-exposed mutants (Fig. 3a). The direct Bmp target, *msx1* in the pharyngeal mesenchyme was also unaffected (Fig. 3b). We found no change in Endothelin ligand (*edn1*) or Endothelin receptor (*ednraa*) expression in ethanol-exposed mutants or heterozygotes (Fig. 3c).

Bmp and End1 signaling regulates the nested expression pattern of genes required for ventral (*hand2*, *dlx5*, *dlx6*) and intermediate (*dlx3*, *dlx4*, *barx1*, *nkx3.2*) arch specification^11,30,31^. We first examined *hand2* expression as it is necessary for lower jaw development. The expression of *hand2* in the mandibular, but not hyoid, arch was lost in 87.5% (7/8) of ethanol-exposed mutants and 22.72% (5/22) of exposed heterozygotes (Fig. 4a). Consistently, *hand2* expression was lost while *bmp4* was maintained in ethanol-treated mutants and heterozygotes, demonstrating that the defect is independent of Bmp (Extended Data Fig. 8). 83.33% (5/6) ethanol-exposed *kat6a* mutants and 11.11% (2/18) exposed heterozygotes lost *dlx5a* (Fig. 4b, Extended Data Fig. 9). Interestingly, only 20% (2/10) *kat6a* mutants lost *dlx6a* expression in the mandibular arch (Fig. 4d) All exposed heterozygotes maintained *dlx6a* expression. Within the intermediate zone, *dlx4b,* but not *dlx3b,* was downregulated in the mandibular arch of 60% (3/5) ethanol-exposed mutants (Fig. 4c). All exposed heterozygotes maintained both *dlx3b* and *dlx4b* expression. The Edn1 targets *dlx5/6* and *dlx3/4* are thought be coregulated^32^ in ventral and intermediate zones respectively. Our results clearly show these pairs are not always coregulated. Concomitant downregulation of *hand2*, *dlx5a* and *dlx4b* results in fusion of palatoquadrate and Meckel’s cartilage^7^, similar to ethanol-exposed *kat6a* mutants (Fig.1c). This supports a model where the defects in ethanol-exposed mutants are caused by loss of expression of these genes.

The expression of other intermediate genes further supports a first arch specific patterning defect. Barx1 is required for the onset of chondrogenesis^33^ and was specifically depleted in the mandibular arch in all (5/5) ethanol-exposed mutants and slightly reduced in 40% (4/10) exposed heterozygotes (Fig. 4e). Nkx3.2 is required for the formation of the primary jaw joint in vertebrates^34^, and its expression is primarily restricted to the mandibular arch in wildtype fish. It is ectopically expressed in the second arch of *kat6a* mutants^14,35^ (arrowhead in Fig. 4f) due to transformation of hyoid into mandibular arch. Interestingly, 80% (4/5) ethanol exposed *kat6a* mutants lost *nkx3.2* expression in the mandibular arch but maintained the ectopic expression in the second arch (arrow in Fig. 4f). These results demonstrate that cell stress results in *kat6a*-dependent disruption of ventral patterning specifically in the mandibular arch (Fig. 4g,h).

We found that dorsal patterning was normal in exposed mutants and heterozygotes. The entire dorsoventral axis of all arches express *dlx2a*^36^ and this expression was unaffected in the dorsal mandibular arch (Fig. 4b). Interestingly, in 33.33% (2/6) ethanol-exposed mutants, *dlx2a* expression was lost in the ventral-most mandibular crest (Extended Data Fig. 9). Dorsal pharyngeal arch specification requires *jagged1b*, encoding a Notch ligand^12^, its expression is unaltered in the mandibular arch of exposed mutants and heterozygotes (Extended Data Fig. 10). Edn1 signaling restricts *jagged1b* expression to the dorsal pharyngeal arch^12,37^. This finding further supports that *kat6a* is acting independent of Edn1/Bmp signaling.

Collectively, these results suggest that exposure in *kat6a* mutants and heterozygotes directly results in a mispatterning of the neural crest within the first pharyngeal arch. We tested this in tissue mosaics by transplanting mandibular crest from wildtype *sox10:mRFP* embryos to ethanol-exposed *kat6a* mutants (Fig. 5a). We found that restoring wild type *kat6a* function in the mandibular arch of ethanol-exposed *kat6a* mutants rescued *hand2* expression in the transplanted side of the embryo. In contrast, the contralateral side was devoid of *hand2* mandibular arch expression (Fig. 5b). Further, we were able to rescue the anterior position of the oral cavity and the epithelial lining of the mouth in tissue mosaics containing wildtype *sox10:mRFP* cells in the mandibular arch of ethanol-exposed *kat6a* mutants (Fig. 5c), demonstrating a neural crest autonomous defect in the oral ectoderm. Tissue mosaics with transplanted *sox10:mRFP* cells in hyoid and posterior arches failed to rescue *hand2* expression in the mandibular arch. This shows that *kat6a* is required autonomously in first arch neural crest mesenchyme for normal jaw patterning in ethanol-exposed embryos.

## Multifactorial interaction of *kat6a* with ethanol and *mef2ca*

The overall pattern of ventral mis-specification closely mirrors those observed in *mef2ca* mutants. Notably, most Bmp/Edn1 targets are lost in the mandibular and hyoid arch, with the exception of *dlx3b*. Also, *mef2ca* mutants show stronger *dlx5a* downregulation than *dlx6a*^38^. These molecular phenotypes are similar to what we observe in exposed *kat6a* mutants and heterozygotes. We previously showed show that *mef2ca* genetically interacts with *edn1* but it is not regulated by *edn1*^38^. Therefore, we postulated that a first arch specific loss of *mef2ca* expression could explain the phenotypes of exposed *kat6a* embryos.

Consistent with our previous findings, we found that *mef2ca* expression starts to emerge in the pharyngeal mesenchyme at 20 hpf^38^. Its expression is uniform along the dorsoventral axis of the mandibular and hyoid arches. However, it is more weakly expressed in the mandibular arch relative to hyoid arch (Extended Data Fig. 11). We found that ethanol had no effect on the initiation of this expression (Extended Data Fig. 11).

Around 28-30 hpf, *mef2ca* expression in the mandibular arch becomes restricted to the anteroventral region, fate mapped to generate Meckel’s cartilage^39^ (Fig. 6a). At 30 hpf, *mef2ca* expression was lost specifically in the mandibular arch of 75% (9/12) ethanol-exposed mutants and 18.18% (6/33) of exposed heterozygotes. There was no observable decrease in *mef2ca* expression in hyoid or posterior arches of ethanol-exposed embryos (Extended Data Fig. 11, Fig. 6a). Further, we found that *mef2ca* and *hand*2 were both absent in the mandibular arch in 80% (4/5) ethanol-exposed *kat6a* mutants (Extended Data Fig. 12). This suggests *kat6a* serves a specific first arch function under conditions of cell stress and defines a critical time window when Mef2ca functions to pattern the ventral arch.

If the loss of *mef2ca* expression is the cause of ventral patterning defects in *kat6a* depleted fish, then knocking down *mef2ca* should phenocopy the effects of exposure. We knocked down *mef2ca* using CRISPR-Cas9 in *kat6a* mutants and heterozygotes. Injection of 1nL CRISPR-Cas9 mix did not disrupt the craniofacial skeleton in wildtypes. This dose resulted in two strong peaks in PCR-high resolution melt (HRM) analysis, suggesting that the fish were largely heterozygous for *mef2ca*. 47.72% (21/44) F0 *mef2ca; kat6a^−/−^* embryos exhibited partial loss of Meckel’s cartilage with no defects in neurocranium or in hyoid and posterior arch elements (Fig. 6b (mild)). Another 11.36% (5/44) F0 *mef2ca; kat6a^−/−^* embryos displayed defects in both Meckel’s cartilage and ventral hyoid arch elements (Fig. 6b (severe)).

We tested the sensitivity of *kat6a* mutants and heterozygotes to *mef2ca* depletion by lowering the CRISPR/Cas9 dose to 0.5 nL. The 0.5nL injected F0 embryos showed no clear mutant peak using HRM, suggesting these fish are genetic mosaics of largely wildtype cells. The hyoid was normal in all F0 *mef2ca; kat6a^−/−^* fish, however, 66.67% (10/15) had partial loss of Meckel’s cartilage (Extended Data Fig. 14a). We found no evidence of an ethanol-*mef2ca* interaction. Both untreated and ethanol-treated F0 *mef2ca;kat6a^+/+^* displayed similar defects (Extended Data Fig. 13).

If the mandibular arch defects are due to disrupted ventral patterning, then we anticipate a concurrent loss of *mef2ca* and specifiers such as *hand2*. We injected 3nL *mef2ca* CRISPR/Cas9 into embryos from crosses of *kat6a* heterozygotes. We confirmed *mef2ca* heterozygosity using HRM and the consequent reduction in *mef2ca* expression using sm-FISH in the F0 embryos. We found that in wildtype un-injected embryos, the mandibular arch had normal *mef2ca* expression, but low compared to other arches as previously observed^33^ (Extended Data Fig. 14b). CRISPR-mediated knockdown further reduced the *mef2ca* expression. This was most notable in the mandibular arch, likely due to its lower baseline level of expression. Importantly, even when *mef2ca* expression was depleted in the mandibular arch of wildtype F0 embryos, *hand2* expression was not completely lost. However, in *kat6a^+/−^* and *kat6a^−/−^* embryos, reduction in *mef2ca* expression resulted in near complete loss of *hand2* in the mandibular arch (Extended Data Fig. 14b). This demonstrates that a combined depletion of *kat6a* and *mef2ca* results in ventral arch patterning defects in the mandibular arch.

We tested the specificity of this interaction by knocking down *edn1*^40^ in *kat6a* mutants and heterozygotes. No additional defects were observed in ethanol-exposed MO-injected wildtypes compared to the unexposed MO-injected wildtypes (Extended Data Fig. 15). Knocking down *edn1* in *kat6a* heterozygotes and mutants did exacerbate the cartilage defects. However, these defects were not restricted to the mandibular arch and do not recapitulate the phenotypes observed in exposed mutants and wildtypes, further supporting a specific effect via *mef2ca*.

## Discussion:

Dorsoventral arch patterning require Bmp/Edn1 signaling from aboral ectoderm and *mef2ca* expression in the pharyngeal arch mesenchyme^11,38^. Bmp/Edn1 and *mef2ca* independently regulate dorsoventral patterning with several common targets within ventral and intermediate arch regions. We show, *mef2ca* expression but not Bmp/Edn1 signaling is susceptible to environmental stressors, such as ethanol, in the mandibular arch. Under cellular stress, *kat6a* is necessary for *mef2ca*-mediated ventral mandibular arch patterning. Absence of *kat6a* in ethanol-exposed mandibular crest results in loss of *mef2ca* and its target genes, *hand2*, *dlx5a*, *dlx6a* and *dlx4b* (Fig. 6c). As previously demonstrated, loss of *mef2ca* does not affect *dlx3b* expression^38^ (Fig. 4c). Edn1 however, promotes *dlx3b* expression^37^, suggesting *dlx3b* might be regulated by Edn1 and not Mef2ca. Conversely, Edn1 and Mef2ca are both required for maintenance of *hand2*, *dlx5a* and *dlx6a* in the ventral arch.

A fundamental finding of this study is uncovering how *kat6a* links anteroposterior and dorsoventral arch patterning, which were previously thought to be independently regulated by distinct genetic pathways. Although the role of *kat6a* in anteroposterior patterning via *hox* gene regulation in hyoid and posterior arches was known^14^. Its requirement in mandibular arch development remained unexplored. In humans, individuals with KAT6A syndrome often present with micrognathia similar to mouse *Kat6a* mutants^2,41^, indicating role of Kat6a in mandibular arch development. Here, we demonstrate its role in ventral mandibular arch patterning, establishing *kat6a* as a bridge between anteroposterior and dorsoventral patterning during pharyngeal arch morphogenesis.

In humans, KAT6A syndrome is highly variable. Studies have hinted at potential genotype-phenotype associations^15^. However, inherited cases of KAT6A syndrome are highly phenotypically variable^19^, suggesting that environmental stressors may generate phenotypic variation. We show that loss of *kat6a* sensitizes embryos to common environmental exposures such as ethanol, nicotine and PBO. Environmental perturbations are often thought to occur via epigenetic modifications^42,43^. Being a chromatin modifier, *Kat6a* itself is an epigenetic regulator. Consequently, the interaction between these external stressors and *kat6a* can produce a magnitude of complex phenotypic variations explaining the variable penetrance in KAT6A syndrome.

We also demonstrate that ethanol-exposed *kat6a* mutants exhibit a selective loss of *mef2ca* expression within the mandibular arch, which disrupts ventral mandibular patterning. Furthermore, we characterize a *kat6a-mef2ca* genetic interaction that provides a second mechanistic layer in both ethanol-exposed *kat6a* mutants and heterozygotes. Interestingly, KAT6A syndrome displays significant phenotypic overlap with both FAS and MEF2C haploinsufficiency syndrome, suggesting a common mechanistic link among these congenital disorders.

## Methods

### 1. Zebrafish husbandry

Zebrafish were raised and housed in the University of Texas at Austin using established protocols^44^. All experiments and procedures were performed as outlined in IACUC protocol AUP-2023-00297 at UT Austin. All embryos were raised at 28.5 °C. *Tg(fli1:EGFP)y1;kat6a^b719^* was acquired from The Crump laboratory, University of Southern California. The *b719* allele has a one nucleotide (cytosine) deletion in exon 14 of *kat6a* and is tightly linked to *fli1:EGFP* on Linkage Group 5^39^. We refer to this line as *kat6a;fli1:GFP*. Genotyping for *kat6a^b719^* was performed using primer set: 5’ GGAGAGGAGGAACGTAAAGAGGTAT 3’ and 5’ AAGAGGATGGTGGAGAGCCTTG 3’ followed by BsaJI restriction digestion to generate 217 bp and 22 bp products in wildtype and 239 bp product in mutants^14^. For sm-FISH experiments, *kat6a^b719^* genotyping was performed using HRM (High resolution melting) analysis with primer sets: 5’ GCTTCCCAGCATTCCCAAAA 3’ and 5’ GGAAGAGGATGGTGGAGAGC 3’ to generate 47 bp of product in wildtype and 46 bp of product in mutants. Migratory NCCs were visualized using *Tg(sox10:mRFP)vu234* line^45^, referred to as *sox10:mRFP* in the text.

### 2. Chemical treatment

Zebrafish *kat6a;fli1:GFP* embryos were exposed to 1% ethanol concentration (v/v) in embryo media (EM) (approx. 41 mM tissue level concentration^46^) from 6-30 hpf for the initial characterization of craniofacial morphology. Embryos were exposed to different ethanol concentrations of 0.125%, 0.25%, 0.5%, 0.75%, 1% v/v in EM from 6-30 hpf for the dose response study. To determine the window of ethanol sensitivity, embryos were exposed to 1% ethanol (v/v) in EM from 6-24 hpf, 8-24 hpf, 9-24 hpf, 10-24 hpf and 6 hpf-4 dpf. Embryos were exposed to 1% ethanol concentration (v/v) in embryo media (EM) from 10-24 hpf to study the effect of *kat6a*-ethanol interaction on DV patterning. Post exposure, the ethanol solution was replaced with EM.

To investigate the effect of Nicotine (Sigma Aldrich, CAS number 65-31-6), 100 mM stock solution was prepared in EM and stored at −20 °C. Zebrafish embryos were exposed to nicotine from 6-24 hpf with a final concentration of 12.5 μM and 25 μM in EM. To investigate the effect of Piperonyl butoxide (PBO, CAS# 51-03-6, Toronto Research Chemicals) a 100mM stock solution was prepared in dimethyl sulfoxide (DMSO) and stored at −20 °C. Zebrafish embryos were exposed to a final concentration of 12.5 μM and 25 μM PBO in EM from 6-24 hpf with control fish at equivalent DMSO dose in EM. Post exposure, the chemical solutions were replaced with EM. At 4 dpf zebrafish were harvested for bone & cartilage staining.

### 3. Alcian-Alizarin (cartilage-bone) double staining

Alcian-blue & alizarin-red (double) staining was performed based on a previously established protocol^47^. Fish were fixed in 2% PFA at room temperature for an hour and stained in alcian-blue solution overnight at room temperature. Fish were rehydrated using ethanol/0.1% KOH solution and bleached using 3% H_2_O_2_ in 0.1% KOH followed by two washes of 25% glycerol/0.1% KOH solution for 10 min each. Fish were then stained in Alizarin red for 30 minutes, followed by washing in 50% glycerol/0.1% KOH solution. Fish were imaged in 50% glycerol/0.1% KOH using a Zeiss Axioimager A1, with a 10X objective lens.

### 4. Whole Mount-Immunohistochemistry for pHH3 staining

Control and ethanol-exposed embryos were dechorionated, then harvested at 28 hpf and fixed overnight in 4% PFA. A pHH3 primary antibody (Cell Signaling, 3377) was used at a 1:500 dilution to detect proliferative cells. Embryo processing and staining was carried out according to a previously described protocol^48^. Embryos were incubated in primary antibodies at 4 °C in 5% normal goat serum, 1%BSA, 1% DMSO, 0.5% Triton X-100. Following washing in PBS with 0.5% Triton X-100, secondary antibody was incubated overnight at 4 °C in 5% normal goat serum, 1% BSA, 1% DMSO, 0.5% Triton X-100 at 1:250 dilution. Secondary antibody incubation was followed by PBS washes and imaging.

### 5. Terminal deoxynucleotidyl transferase dUTP nick end-labeling (TUNEL) staining

Embryos were dechorionated and harvested at 28 hpf and fixed overnight in 4% PFA. Apoptosis was analysed using the Biotium CF640R TUNEL Assay Apoptosis Detection Kit. Embryos were permeabilized using a 25 μg/ml solution of proteinase K in 0.5% Tween 20 in PBS for 8 min. The staining protocol was performed following manufacturer's instructions.

### 6. Single molecule-Fluorescent *in-situ* hybridization (sm-FISH)

Whole mount *in situ* hybridization was carried out according to protocol described by Molecular Instruments based on hybridization chain reaction (HCR)^49^. All probes were manufactured by Molecular Instruments.

### 7. Generation of zebrafish mosaics

Transplantation experiments targeting cranial neural crest were performed as previously described^50,51^. *kat6a;fli1:GFP* and *sox10:mRFP* embryos were collected and allowed to develop at 28.5 °C and dechorionated at 4 hpf. At shield stage (6 hpf) cells from donor embryo (*sox10:mRFP*) were transplanted targeting the neural crest progenitor domain in host embryos (*kat6a;fli1:GFP*) as previously described^52^. Transplanted embryos were allowed to develop until bud stage (10 hpf). EM was replaced with 1% ethanol (v/v) in EM. At 24 hpf, embryos were transferred to normal EM and embryos with transplants targeting the first or second arches were imaged via confocal. Embryos were harvested at 28 hpf for *in situ* hybridization or allowed to develop to 4 dpf for investigation of oral cavities and cranial cartilages. Oral cavity defects were studied using the brightfield channel of confocal images. Imaged fish were then individually stained using alcian-alizarin staining protocols to characterize skeletal defects.

### 8. CRISPR-mediated knockdown

*mef2ca* gRNA (IDT) was suspended in 20 μl of duplex buffer (IDT). TracrRNA (IDT) was resuspended in 50 μl of duplex Buffer to obtain a final concentration of 100 μM and stored at −20°C. CRISPR mix containing 0.6 μl of 2% Phenyl red, 0.5 μl of gRNA suspended in duplex buffer, 0.5μl of 100μM TracrRNA, 0.5 μl of Alt-R S.p. HiFi Cas9 nuclease (IDT) and 3.9 μl of nuclease free water was freshly prepared before injections based on previously described protocol^53^ .Embryos were injected at the one cell stage with either 0.5, 1 or 3 nl and CRISPR solution. They were either harvested at 28 hpf for *in-situ* hybridization or allowed to develop until 4 dpf for alcian-alizarin staining.

### 9. High Resolution Melt (HRM) analysis

Genotyping using HRM^54^ was performed using 12 μl reaction mix containing, 6 μL of MeltDoctor HRM Master mix (Fisher Scientific 4415450), 0.3 μM primer pair and 1μl of genomic DNA. Melt curves were generated using Thermofisher QuantStudio 3 and analysed using Thermofisher HRM software. Distinct peaks for mutants and wild types were generated and heterozygotes displayed double peaks. Two separate primer pairs were used to confirm the *mef2ca* CRISPR injected F0 fish with double peaks: 5’ GCACGAGAGTCGGACTAATTC 3’ and 5’ AACAGGAACTGAGCTGGAAC 3’ for 58 bp amplicon in wildtypes. 5’ TGCTGAAATACACCGAGTACAA 3’ and 5’ CAACAGGAACTGAGCTGGAA 3’ for 87 bp amplicon in wildtypes.

### 10. Quantitative reverse transcription PCR (RT-qPCR)

RT-qPCR was performed to investigate the effect of ethanol on *kat6a* transcriptional expression at 30 hpf. Embryos were treated with ethanol from 6-24 hpf. GFP-negative, wildtype *kat6a* siblings were sorted using the linkage of *fli1:GFP* with the *kat6a*^719^ mutation^39^. Total RNA was extracted from 7 wildtype embryos at 30 hpf in control and ethanol-treated group in triplicates using TRIzol RNA isolation protocol. Total RNA was reverse transcribed using SuperScript II First-Strand Synthesis System (Invitrogen) with oligo-d(T) primers. A standard curve using 0, 1.25, 2.5, 5, 10 and 20 ng of cDNA was generated to determine optimum cDNA concentration for qPCR reaction. RT-qPCR was performed with 5 ng of cDNA using Power Sybr Green PCR Master Mix (Thermo Fisher Scientific, 4367659) on the Applied Biosystems ViiA^™^ 7 Real-Time PCR System. Raw data obtained from QuantStudio 3 Real-Time PCR Software was analysed using 2-ΔΔCt method for relative gene expression. Endogenous controls were *solute carrier family 25 (slc25a5)* and *like-Sm protein 12 homolog b (lsm12b)* based on previous findings from our lab showing their stable expression in untreated and ethanol-treated fish across developmental stages^55^.

*kat6a*^719^ primer pairs: 5' CTGGTAAGCAGGAGTTCTCAC 3' and 5' AACAGAAACTACAGATGGGTATAGG 3'

*slc25a5* primer pairs: 5' ACACAGCCAAGGGTATGCTG 3’ and 5’ GTGTCGAAGGGGTAGGAAGC 3’

*lsm12b* primer pairs: 5’ AGTTGTCCCAAGCCTATGCAATCAG 3’ and 5’ CCACTCAGGAGGATAAAGACGAGTC 3’

### 11. Morpholino injection for *edn1* knockdown

A previously described morpholino against *edn1* was used^56^. Approximately 1 nl of morpholino solution (final concentration 3 mg/ml) with phenol red dye was injected into zebrafish embryos at the one-cell and stage. At bud stage, EM was replaced with 1% ethanol (v/v) in EM in the experimental dish and allowed to develop until 4 dpf. At 4dpf control and experimental fish were stained with alcian-alizarin.

### 12. Confocal imaging

Zebrafish were anesthetized and mounted in 0.2% agarose on 3% Methyl Cellulose. Imaging was carried out based on previously described protocol^57^ using Zeiss LSM 710 & LSM 980 laser scanning microscopes. All confocal images were processed using FIJI/ImageJ software.

### 13. Statistical Analyses

Fisher’s exact test of independence (two-sided) was used to determine if the effect of ethanol at different developmental windows on Meckel’s cartilage was statistically significant. Fisher’s exact test of independence (two-sided) was also used for ethanol dose response study. Unpaired t-test was used to compare the level of *kat6a* expression in untreated and ethanol-treated wildtype embryos using RT-qPCR. Two-way ANOVA with Tukey’s multiple comparisons correction was used to determine if ethanol affects the length or area measurements of cartilaginous elements, apoptosis and proliferation of neural crest cells in *kat6a* mutants and heterozygotes. Data are presented as means ± SEM and p < 0.05 was considered statistically significant. GraphPad Prism was used for all statistical analyses.

## Supplementary Material

1

## Figures and Tables

**Fig.1∣:**
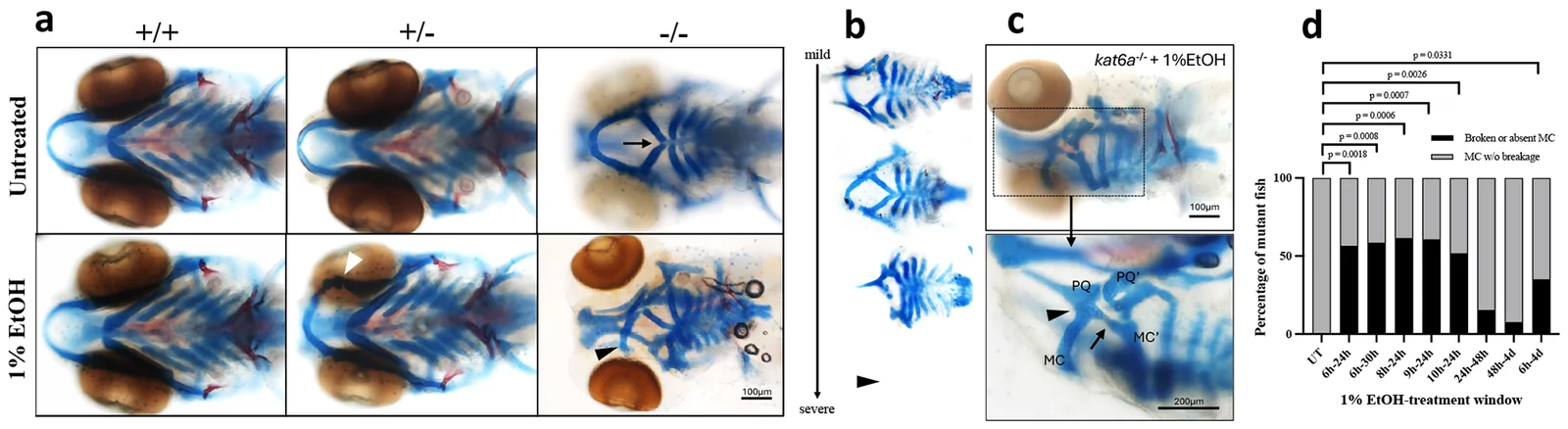
Ethanol induces jaw defects in *kat6a* mutants and heterozygotes **a,** Double stained, untreated and ethanol-treated *kat6a* wildtypes, heterozygotes and mutants at 4dpf. Arrow shows transformed ceratohyals mirroring Meckel’s cartilage in untreated mutant. Black arrowhead indicates broken Meckel’s cartilage in an ethanol exposed mutant. White arrowhead indicates mandibular skeletal defects in ethanol exposed heterozygotes **b,** Flat mounts of viscerocrania illustrating the range of severity in jaw defects in ethanol exposed mutants. Arrowhead indicates complete loss of Meckel’s cartilage in the most severely affected exposed mutant. **c,** Lateral view showing fused Meckel’s cartilage and palatoquadrate in an exposed mutant. Arrowhead indicates lack of jaw joint. Arrow indicates attachment of Meckel’s cartilage to posterior end of ceratohyal (transformed Meckel’s cartilage) **d,** Quantification of mutants with defects in Meckel’s cartilage when exposed to ethanol at various developmental windows between 6h (hours post fertilization) to 4d (days post fertilization). MC (Meckel’s cartilage), PQ (Palatoquadrate), MC’(duplicated Meckel’s cartilage), PQ’ (duplicated Palatoquadrate).

**Fig.2∣:**
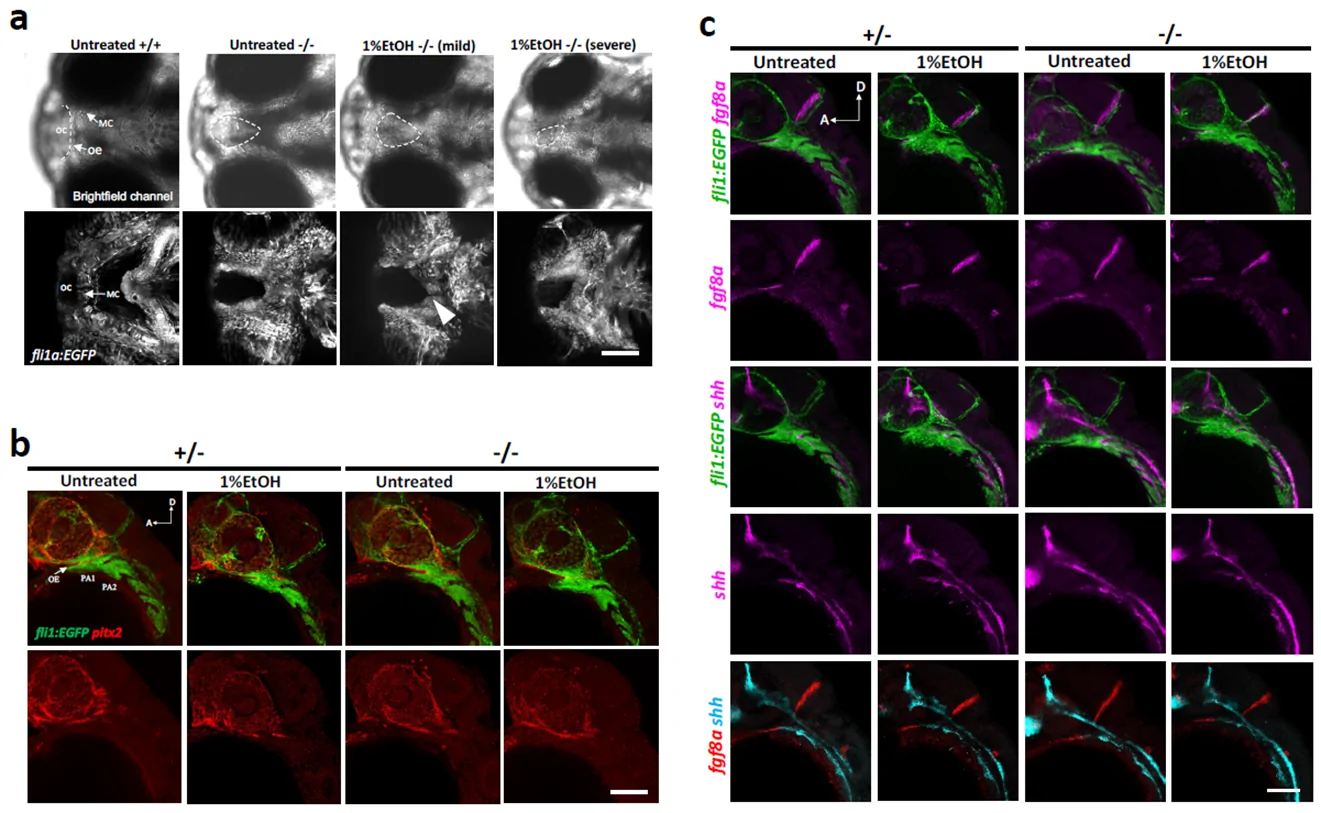
Ethanol does not affect pharyngeal epithelial signaling domains in *kat6a* mutants **a,** Confocal images of ventrally mounted *kat6a;fli1:EGFP* at 4 dpf. Brightfield channel shows oral cavity (oc) in stippled line. *fli1:EGFP* shows the anterior extent of the mandibular arch mesenchyme and Meckel’s cartilage. Arrowhead indicates mispatterned oral cavity along the mid-line. **b,** Lateral view of 28 hpf untreated and ethanol-treated *kat6a;fli1:EGFP* showing *pitx2* (*paired-like homeodomain 2*) expression. **c,** Lateral view of 28 hpf untreated and ethanol-treated *kat6a;fli1:EGFP* showing *shha* (*sonic hedgehog signaling molecule a*) and *fgf8a* (*fibroblast growth factor 8a*) expression. MC (Meckel’s cartilage), oe (oral epithelium), oc (oral cavity), PA (pharyngeal arch), A (Anterior), D (Dorsal). Scale bar = 100 μm

**Fig.3∣:**
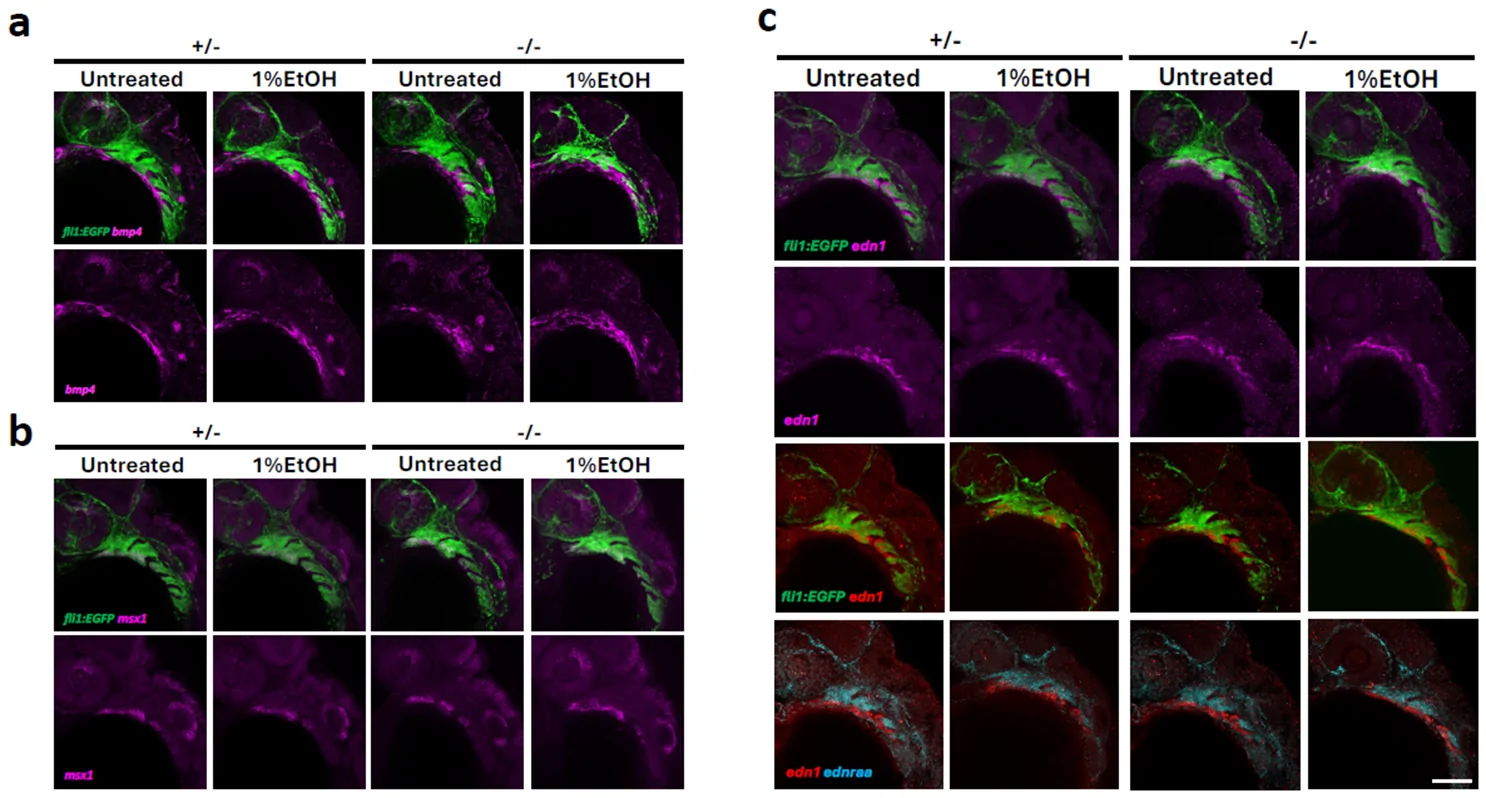
Ethanol does not affect ventral ectodermal signaling in *kat6a* mutants **a,** Untreated and ethanol-treated *kat6a;fli1:EGFP* showing normal *bmp4* (*bone morphogenetic protein 4*) expression. **b,** Untreated and ethanol-treated *kat6a;fli1:EGFP* showing normal *msx1a* (*muscle segment homeobox 1a*) expression. **c,** Untreated and ethanol-treated *kat6a;fli1:EGFP* showing normal *edn1* (*endothelin 1*) and *ednraa* (*endothelin receptor type Aa*) expression. All images are lateral views at 28 hpf. Scale bar = 100 μm

**Fig.4∣:**
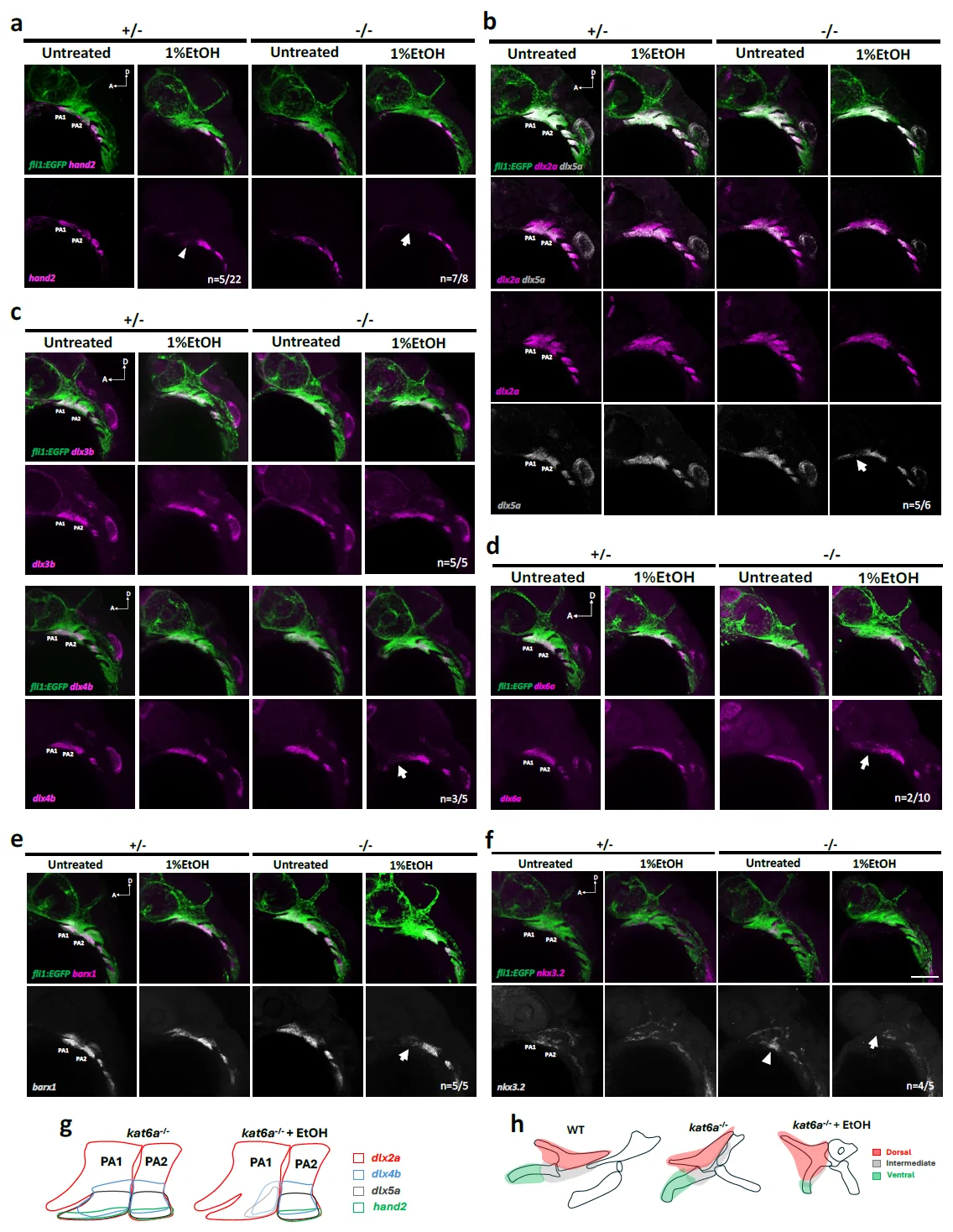
Ethanol induces ventral patterning defects in mandibular crest of *kat6a* mutants and heterozygotes **a-f** show lateral views of untreated and ethanol-treated, *kat6a;fli1:EGFP* at 28 hpf. **a,** Embryos with *hand2* (*heart and neural crest derivatives expressed 2*) expression show loss of *hand2* in mandibular arch of an exposed mutant (arrow) and heterozygote (arrowhead). **b,** Embryos with *dlx* (*distal-less homeobox*) *2a* and *5a* expression showing loss of *dlx5a* (arrow) in the mandibular arch of an exposed mutant. **c,** Embryos with *dlx3b* and *dlx4b* expression showing loss of *dlx4b* (arrow) in the mandibular arch of an exposed mutant. **d,** Embryos with *dlx6a* expression showing loss of *dlx6a* in the mandibular arch of an exposed mutant (arrow). **e,** Embryos with *barx1* (*BARX homeobox 1*) expression showing loss of *barx1* in the mandibular arch of an exposed mutant (arrow). **f,** Embryos with *nkx3.2* (*nk3 homeobox 2*) expression with loss of *nkx3.2* in the mandibular arch of an exposed mutant (arrow). Arrowhead indicates elevated expression of *nkx3.2* in an unexposed mutant. **g,** Schematic showing downregulation of *dlx4b*, *dlx5a* and *hand2* in the mandibular but not the hyoid arch of ethanol-exposed *kat6a* mutants. **h,** Schematic showing lateral view of mandibular and hyoid arch elements in wildtypes, *kat6a* mutants and ethanol-exposed *kat6a* mutants. Loss of *kat6a* affects hyoid arch patterning. Ethanol exposure reduces the ventral and intermediate domains with loss of jaw joint in mutants. Scale bar = 100 μm

**Fig.5∣:**
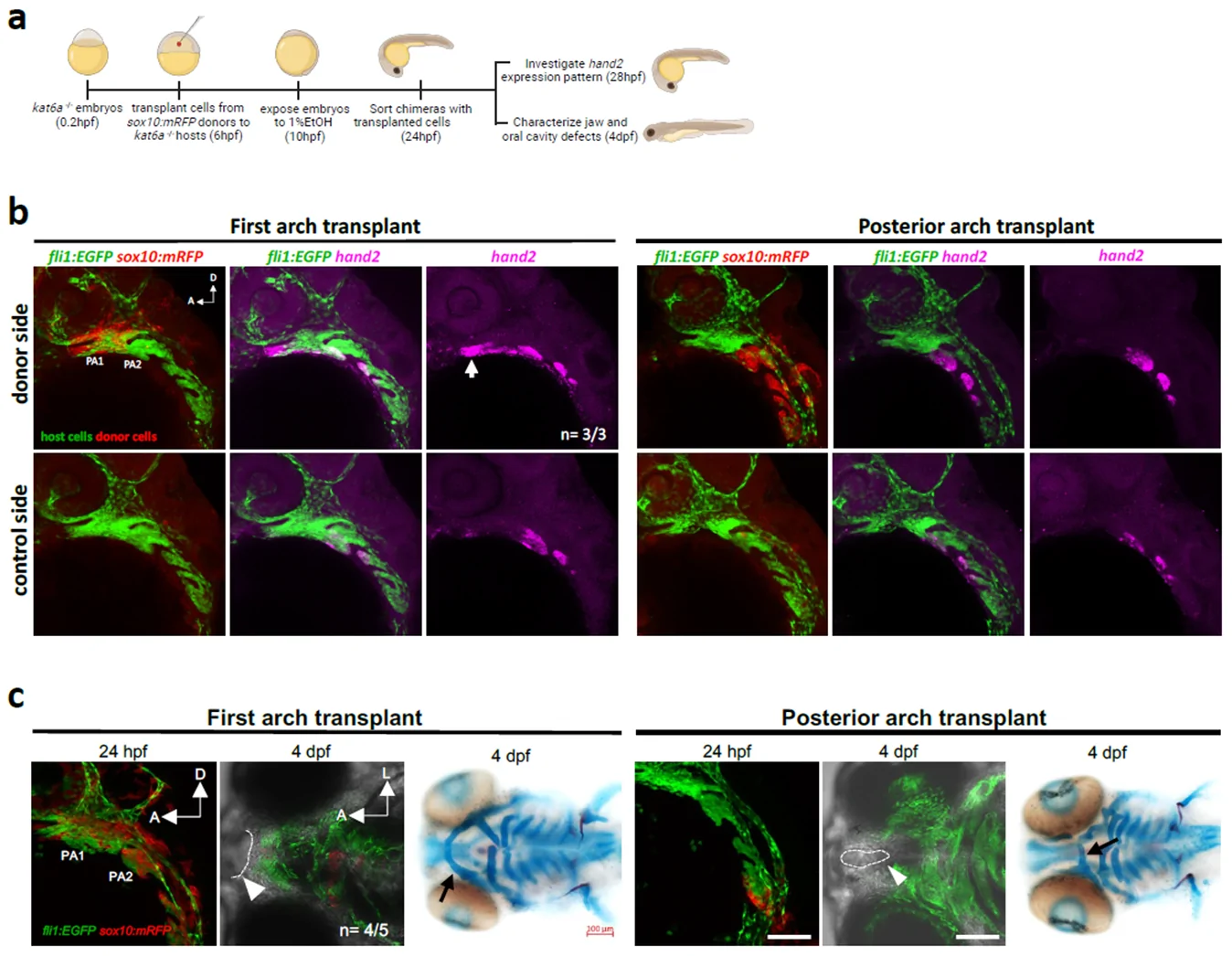
Mandibular neural crest requires *kat6a* for neural crest patterning and oral ectoderm morphogenesis. **a,** Timeline for generation and analysis of chimeras (created using BioRender). **b,** Lateral views of donor (transplanted) and control sides of ethanol-exposed *kat6a* mutants with wildtype neural crest cells from *sox10:mRPF* in mandibular (first) arch or posterior arches at 28 hpf. Arrow indicates rescued *hand2* expression in the donor side with wildtype, *sox10* expressing neural crest cells in the mandibular arch. The expression of *hand2* remains lost on the control side of the embryo or in embryos with neural crest cells populating more posterior arches. **c,** Lateral (donor side at 24 hpf) and ventral views (at 4 dpf) of mandibular arch or posterior arch-specific chimeras. White arrow in first arch transplant shows rescued oral cavity anteriorly located (stippled line). White arrow in posterior arch transplant shows malformed and posteriorly located oral cavity (stippled line). Black arrow shows double stained rescued Meckel’s cartilage in first arch transplant but not in posterior arch transplant. Scale bar = 100 μm

**Fig.6∣:**
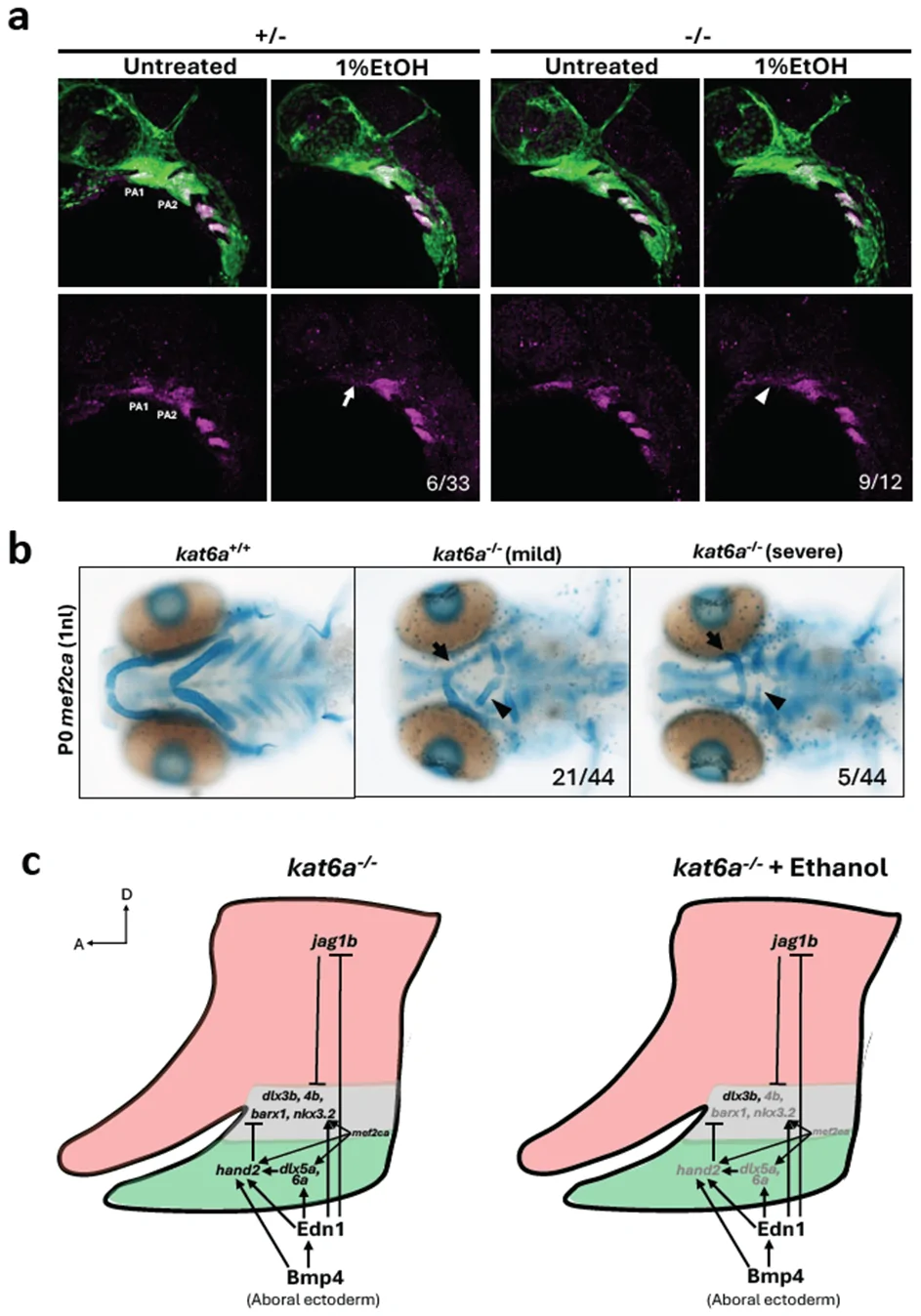
Ethanol-induced loss of *mef2ca* expression in the mandibular arch results in ventral mis-patterning **a.** Lateral view of *kat6a;fli1a:EGFP* at 28 hpf stained for *mef2ca* expression. Arrow shows loss of *mef2ca* expression in mandibular arch of ethanol exposed heterozygotes. Arrowhead shows loss of expression in exposed mutant. **b,** 1nL *mef2ca* CRISPR-injected *kat6a* wildtype and mutants. Mild phenotype showed partial loss of Meckel’s cartilage (arrow) with no loss of hyoid or posterior arch skeleton (arrowhead). Severe phenotype showed severely affected Meckel’s cartilage (arrow) and hyoid skeleton (arrowhead). **c,** Schematic showing effect of ethanol on a gene network required for dorsoventral patterning. Loss of *kat6a* has no impact on the gene network in unexposed embryos. Critical components for ventral specification are lost or disrupted (labelled in grey) following ethanol exposure.

## Data Availability

All numerical and statistical data are provided in supplementary data and supplementary tables, and where possible mentioned in the manuscript text. The data that support the findings of this study are available upon reasonable request from the corresponding authors, RG & JKE.

## References

[1] Zweier M., & Rauch A. (2012). The MEF2C-Related and 5q14.3q15 Microdeletion Syndrome. Molecular syndromology, 2(3-5), 164–170. 10.1159/000337496 PMC3366707 22670137

[2] Tham E., Lindstrand A., Santani A., Malmgren H., Nesbitt A., Dubbs H. A., Zackai E. H., Parker M. J., Millan F., Rosenbaum K., Wilson G. N., & Nordgren A. (2015). Dominant mutations in KAT6A cause intellectual disability with recognizable syndromic features. American journal of human genetics, 96(3), 507–513. 10.1016/j.ajhg.2015.01.016 PMC4375419 25728777

[3] Cooley Coleman J, Skinner SA. MEF2C-Related Disorder. 2024 Dec 12. In: Adam MP, Bick S, Mirzaa GM, , editors. GeneReviews^®^ [Internet]. Seattle (WA): University of Washington, Seattle; 1993-2026. Available from: https://www.ncbi.nlm.nih.gov/books/NBK610216/39666846

[4] Muggli E., Matthews H., Penington A., Claes P., O'Leary C., Forster D., Donath S., Anderson P. J., Lewis S., Nagle C., Craig J. M., White S. M., Elliott E. J., & Halliday J. (2017). Association Between Prenatal Alcohol Exposure and Craniofacial Shape of Children at 12 Months of Age. JAMA pediatrics, 171(8), 771–780. 10.1001/jamapediatrics.2017.0778 PMC6583660 28586842

[5] Barrow J. R., & Capecchi M. R. (1999). Compensatory defects associated with mutations in Hoxa1 restore normal palatogenesis to Hoxa2 mutants. Development (Cambridge, England), 126(22), 5011–5026. 10.1242/dev.126.22.5011 10529419

[6] Minoux M., Antonarakis G. S., Kmita M., Duboule D., & Rijli F. M. (2009). Rostral and caudal pharyngeal arches share a common neural crest ground pattern. Development (Cambridge, England), 136(4), 637–645. 10.1242/dev.028621 PMC4482666 19168678

[7] Talbot J. C., Johnson S. L., & Kimmel C. B. (2010). hand2 and Dlx genes specify dorsal, intermediate and ventral domains within zebrafish pharyngeal arches. Development (Cambridge, England), 137(15), 2507–2517. 10.1242/dev.049700 PMC2927700 20573696

[8] Swartz M. E., Nguyen V., McCarthy N. Q., & Eberhart J. K. (2012). Hh signaling regulates patterning and morphogenesis of the pharyngeal arch-derived skeleton. Developmental biology, 369(1), 65–75. 10.1016/j.ydbio.2012.05.032 PMC3413459 22709972

[9] Gendron-Maguire M., Mallo M., Zhang M., & Gridley T. (1993). Hoxa-2 mutant mice exhibit homeotic transformation of skeletal elements derived from cranial neural crest. Cell, 75(7), 1317–1331. 10.1016/0092-8674(93)90619-2 7903600

[10] Hunter M. P., & Prince V. E. (2002). Zebrafish hox paralogue group 2 genes function redundantly as selector genes to pattern the second pharyngeal arch. Developmental biology, 247(2), 367–389. 10.1006/dbio.2002.0701 12086473

[11] Alexander C., Zuniga E., Blitz I. L., Wada N., Le Pabic P., Javidan Y., Zhang T., Cho K. W., Crump J. G., & Schilling T. F. (2011). Combinatorial roles for BMPs and Endothelin 1 in patterning the dorsal-ventral axis of the craniofacial skeleton. Development (Cambridge, England), 138(23), 5135–5146. 10.1242/dev.067801 PMC3210495 22031543

[12] Barske L., Askary A., Zuniga E., Balczerski B., Bump P., Nichols J. T., & Crump J. G. (2016). Competition between Jagged-Notch and Endothelin1 Signaling Selectively Restricts Cartilage Formation in the Zebrafish Upper Face. PLoS genetics, 12(4), e1005967. 10.1371/journal.pgen.1005967 PMC4825933 27058748

[13] Voss A. K., Vanyai H. K., Collin C., Dixon M. P., McLennan T. J., Sheikh B. N., Scambler P., & Thomas T. (2012). MOZ regulates the Tbx1 locus, and Moz mutation partially phenocopies DiGeorge syndrome. Developmental cell, 23(3), 652–663. 10.1016/j.devcel.2012.07.010 PMC3442180 22921202

[14] Miller C. T., Maves L., & Kimmel C. B. (2004). moz regulates Hox expression and pharyngeal segmental identity in zebrafish. Development (Cambridge, England), 131(10), 2443–2461. 10.1242/dev.01134 15128673

[15] Kennedy J., Goudie D., Blair E., Chandler K., Joss S., McKay V., Green A., Armstrong R., Lees M., Kamien B., Hopper B., Tan T. Y., Yap P., Stark Z., Okamoto N., Miyake N., Matsumoto N., Macnamara E., Murphy J. L., McCormick E., … Newbury-Ecob R. (2019). KAT6A Syndrome: genotype-phenotype correlation in 76 patients with pathogenic KAT6A variants. Genetics in medicine : official journal of the American College of Medical Genetics, 21(4), 850–860. 10.1038/s41436-018-0259-2 PMC6634310 30245513

[16] Bergamasco M. I., Yang Y., Garnham A. L., Sheikh B. N., Smyth G. K., Voss A. K., & Thomas T. (2025). KAT6B overexpression rescues embryonic lethality in homozygous null KAT6A mice restoring vitality and normal lifespan. Nature communications, 16(1), 1958. 10.1038/s41467-025-57155-4 PMC11861323 40000651

[17] Hong M., & Krauss R. S. (2017). Ethanol itself is a holoprosencephaly-inducing teratogen. PloS one, 12(4), e0176440. 10.1371/journal.pone.0176440 PMC5404885 28441416

[18] Manikandan P., Sarmah S., & Marrs J. A. (2022). Ethanol Effects on Early Developmental Stages Studied Using the Zebrafish. Biomedicines, 10(10), 2555. 10.3390/biomedicines10102555 PMC9599251 36289818

[19] Ringsted S. B., Markholt S., Andreasen L., & Gregersen P. A. (2025). Phenotypic variability in a family with an inherited KAT6A frameshift variant. European journal of medical genetics, 73, 104993. 10.1016/j.ejmg.2024.104993 39740728

[20] Gomes G, Andrade ACS, Vieira PC, Spineli MN, Costa ML, Mermelstein C. DMSO induces major morphological and physiological alterations in zebrafish embryos. PLoS One. 2025 Aug 18;20(8):e0330348. doi: 10.1371/journal.pone.0330348. PMC12360551 40824954

[21] May P. A., Baete A., Russo J., Elliott A. J., Blankenship J., Kalberg W. O., Buckley D., Brooks M., Hasken J., Abdul-Rahman O., Adam M. P., Robinson L. K., Manning M., & Hoyme H. E. (2014). Prevalence and characteristics of fetal alcohol spectrum disorders. Pediatrics, 134(5), 855–866. 10.1542/peds.2013-3319 PMC4210790 25349310

[22] May P. A., Chambers C. D., Kalberg W. O., Zellner J., Feldman H., Buckley D., Kopald D., Hasken J. M., Xu R., Honerkamp-Smith G., Taras H., Manning M. A., Robinson L. K., Adam M. P., Abdul-Rahman O., Vaux K., Jewett T., Elliott A. J., Kable J. A., Akshoomod N., … Hoyme H. E. (2018). Prevalence of Fetal Alcohol Spectrum Disorders in 4 US Communities. JAMA, 319(5), 474–482. 10.1001/jama.2017.21896 PMC5839298 29411031

[23] McCarthy N., Wetherill L., Lovely C. B., Swartz M. E., Foroud T. M., & Eberhart J. K. (2013). Pdgfra protects against ethanol-induced craniofacial defects in a zebrafish model of FASD. Development (Cambridge, England), 140(15), 3254–3265. 10.1242/dev.094938 PMC3931738 23861062

[24] Ghosal R., Borrego-Soto G., & Eberhart J. K. (2023). Embryonic ethanol exposure disrupts craniofacial neuromuscular integration in zebrafish larvae. Frontiers in physiology, 14, 1131075. 10.3389/fphys.2023.1131075 PMC9941677 36824468

[25] McCarthy N., Sidik A., Bertrand J. Y., & Eberhart J. K. (2016). An Fgf-Shh signaling hierarchy regulates early specification of the zebrafish skull. Developmental biology, 415(2), 261–277. 10.1016/j.ydbio.2016.04.005 PMC4967541 27060628

[26] Ahsan K., Singh N., Rocha M., Huang C., & Prince V. E. (2019). Prickle1 is required for EMT and migration of zebrafish cranial neural crest. Developmental biology, 448(1), 16–35. 10.1016/j.ydbio.2019.01.018 PMC6480417 30721665

[27] Zbasnik N., & Fish J. L. (2023). Fgf8 regulates first pharyngeal arch segmentation through pouch-cleft interactions. Frontiers in cell and developmental biology, 11, 1186526. 10.3389/fcell.2023.1186526 PMC10242020 37287454

[28] Balczerski B., Matsutani M., Castillo P., Osborne N., Stainier D. Y., & Crump J. G. (2012). Analysis of sphingosine-1-phosphate signaling mutants reveals endodermal requirements for the growth but not dorsoventral patterning of jaw skeletal precursors. Developmental biology, 362(2), 230–241. 10.1016/j.ydbio.2011.12.010 PMC3265674 22185793

[29] Couly G., Creuzet S., Bennaceur S., Vincent C., & Le Douarin N. M. (2002). Interactions between Hox-negative cephalic neural crest cells and the foregut endoderm in patterning the facial skeleton in the vertebrate head. Development (Cambridge, England), 129(4), 1061–1073. 10.1242/dev.129.4.1061 11861488

[30] Alexander C., Piloto S., Le Pabic P., & Schilling T. F. (2014). Wnt signaling interacts with bmp and edn1 to regulate dorsal-ventral patterning and growth of the craniofacial skeleton. PLoS genetics, 10(7), e1004479. 10.1371/journal.pgen.1004479 PMC4109847 25058015

[31] Tavares A. L., Garcia E. L., Kuhn K., Woods C. M., Williams T., & Clouthier D. E. (2012). Ectodermal-derived Endothelin1 is required for patterning the distal and intermediate domains of the mouse mandibular arch. Developmental biology, 371(1), 47–56. 10.1016/j.ydbio.2012.08.003 PMC3470875 22902530

[32] Ghanem N., Jarinova O., Amores A., Long Q., Hatch G., Park B. K., Rubenstein J. L., & Ekker M. (2003). Regulatory roles of conserved intergenic domains in vertebrate Dlx bigene clusters. Genome research, 13(4), 533–543. 10.1101/gr.716103 PMC430168 12670995

[33] Nichols J. T., Pan L., Moens C. B., & Kimmel C. B. (2013). barx1 represses joints and promotes cartilage in the craniofacial skeleton. Development (Cambridge, England), 140(13), 2765–2775. 10.1242/dev.090639 PMC3678344 23698351

[34] Miyashita T., Baddam P., Smeeton J., Oel A. P., Natarajan N., Gordon B., Palmer A. R., Crump J. G., Graf D., & Allison W. T. (2020). nkx3.2 mutant zebrafish accommodate jaw joint loss through a phenocopy of the head shapes of Paleozoic jawless fish. The Journal of experimental biology, 223(Pt 15), jeb216945. 10.1242/jeb.216945 PMC10668335 32527964

[35] Laue K, Daujat S, Crump JG, Plaster N, Roehl HH; Tübingen 2000 Screen Consortium; Kimmel CB, Schneider R, Hammerschmidt M. The multidomain protein Brpf1 binds histones and is required for Hox gene expression and segmental identity. Development. 2008 Jun;135(11):1935–46. doi: 10.1242/dev.017160. PMC2919486 18469222

[36] Sperber S. M., Saxena V., Hatch G., & Ekker M. (2008). Zebrafish dlx2a contributes to hindbrain neural crest survival, is necessary for differentiation of sensory ganglia and functions with dlx1a in maturation of the arch cartilage elements. Developmental biology, 314(1), 59–70. 10.1016/j.ydbio.2007.11.005 18158147

[37] Zuniga E., Rippen M., Alexander C., Schilling T. F., & Crump J. G. (2011). Gremlin 2 regulates distinct roles of BMP and Endothelin 1 signaling in dorsoventral patterning of the facial skeleton. Development (Cambridge, England), 138(23), 5147–5156. 10.1242/dev.067785 PMC3210496 22031546

[38] Miller C. T., Swartz M. E., Khuu P. A., Walker M. B., Eberhart J. K., & Kimmel C. B. (2007). mef2ca is required in cranial neural crest to effect Endothelin1 signaling in zebrafish. Developmental biology, 308(1), 144–157. 10.1016/j.ydbio.2007.05.018 PMC2148033 17574232

[39] Crump J. G., Swartz M. E., Eberhart J. K., & Kimmel C. B. (2006). Moz-dependent Hox expression controls segment-specific fate maps of skeletal precursors in the face. Development (Cambridge, England), 133(14), 2661–2669. 10.1242/dev.02435 16774997

[40] Miller C. T., Yelon D., Stainier D. Y., & Kimmel C. B. (2003). Two endothelin 1 effectors, hand2 and bapx1, pattern ventral pharyngeal cartilage and the jaw joint. Development (Cambridge, England), 130(7), 1353–1365. 10.1242/dev.00339 12588851

[41] Vanyai H. K., Garnham A., May R. E., McRae H. M., Collin C., Wilcox S., Smyth G. K., Thomas T., & Voss A. K. (2019). MOZ directs the distal-less homeobox gene expression program during craniofacial development. Development (Cambridge, England), 146(14), dev175042. 10.1242/dev.175042 31340933

[42] Volkow N. D. (2011). Epigenetics of nicotine: another nail in the coughing. Science translational medicine, 3(107), 107ps43. 10.1126/scitranslmed.3003278 PMC3492949 22049068

[43] Shukla S. D., & Aroor A. R. (2006). Epigenetic effects of ethanol on liver and gastrointestinal injury. World journal of gastroenterology, 12(33), 5265–5271. 10.3748/wjg.v12.i33.5265 PMC4088190 16981253

[44] Westerfield M. (2000). The zebrafish book. A guide for the laboratory use of zebrafish (Danio rerio). 4th Edition. Eugene: University of Oregon Press.

[45] Kucenas S., Takada N., Park H. C., Woodrud E., Broadie K., & Appel B. (2008). CNS-derived glia ensheath peripheral nerves and mediate motor root development. Nature neuroscience, 11(2), 143–151. 10.1038/nn2025 PMC2657597 18176560

[46] Lovely C. B. Nobles R. D. Eberhart J. K. (2014). Developmental age strengthens barriers to ethanol accumulation in zebrafish. Alcohol 48 (6), 595–602. 10.1016/j.alcohol.2014.06.003 PMC4163099 25012627

[47] Walker M. B., & Kimmel C. B. (2007). A two-color acid-free cartilage and bone stain for zebrafish larvae. Biotechnic & histochemistry : official publication of the Biological Stain Commission, 82(1), 23–28. 10.1080/10520290701333558 17510811

[48] Ghosal R., & Eberhart J. K. (2025). Quantification of Neuromuscular Junctions in Zebrafish Cranial Muscles. Bio-protocol, 15(4), e5219. 10.21769/BioProtoc.5219 PMC11865821 40028023

[49] Choi H. M. T., Schwarzkopf M., Fornace M. E., Acharya A., Artavanis G., Stegmaier J., Cunha A., & Pierce N. A. (2018). Third-generation in situ hybridization chain reaction: multiplexed, quantitative, sensitive, versatile, robust. Development (Cambridge, England), 145(12), dev165753. 10.1242/dev.165753 PMC6031405 29945988

[50] Maves L., Jackman W., & Kimmel C. B. (2002). FGF3 and FGF8 mediate a rhombomere 4 signaling activity in the zebrafish hindbrain. Development (Cambridge, England), 129(16), 3825–3837. 10.1242/dev.129.16.3825 12135921

[51] Eberhart J. K., Swartz M. E., Crump J. G., & Kimmel C. B. (2006). Early Hedgehog signaling from neural to oral epithelium organizes anterior craniofacial development. Development (Cambridge, England), 133(6), 1069–1077. 10.1242/dev.02281 16481351

[52] Sato M., & Yost H. J. (2003). Cardiac neural crest contributes to cardiomyogenesis in zebrafish. Developmental biology, 257(1), 127–139. 10.1016/s0012-1606(03)00037-x 12710962

[53] Burger A., Lindsay H., Felker A., Hess C., Anders C., Chiavacci E., Zaugg J., Weber L. M., Catena R., Jinek M., Robinson M. D., & Mosimann C. (2016). Maximizing mutagenesis with solubilized CRISPR-Cas9 ribonucleoprotein complexes. Development (Cambridge, England), 143(11), 2025–2037. 10.1242/dev.134809 27130213

[54] Samarut É., Lissouba A., & Drapeau P. (2016). A simplified method for identifying early CRISPR-induced indels in zebrafish embryos using High Resolution Melting analysis. BMC genomics, 17, 547. 10.1186/s12864-016-2881-1 PMC4973544 27491876

[55] Sidik A., Dixon G., Buckley D. M., Kirby H. G., Sun S., & Eberhart J. K. (2021). Exposure to ethanol leads to midfacial hypoplasia in a zebrafish model of FASD via indirect interactions with the Shh pathway. BMC biology, 19(1), 134. 10.1186/s12915-021-01062-9 PMC8247090 34210294

[56] Miller C. T., & Kimmel C. B. (2001). Morpholino phenocopies of endothelin 1 (sucker) and other anterior arch class mutations. Genesis (United States), 30(3), 186–187. 10.1002/gene.1061 11477704

[57] McGurk P. D., Lovely C. B., & Eberhart J. K. (2014). Analyzing craniofacial morphogenesis in zebrafish using 4D confocal microscopy. Journal of visualized experiments : JoVE, (83), e51190. 10.3791/51190 PMC4089438 24514435

