# Supplementary material for: Kat6a functions to protect lower jaw patterning during cellular stress
Source: Res Sq. 2026 Oct 6:rs.3.rs-10009504. Preprint. [Version 1] doi: 10.21203/rs.3.rs-10009504/v1 (PMC13692630; doi:10.21203/rs.3.rs-10009504/v1)
Supplement: Supplementary file 1 [file NIHPPrs10009504v1-supplement-1.pdf]

Supplementary Data

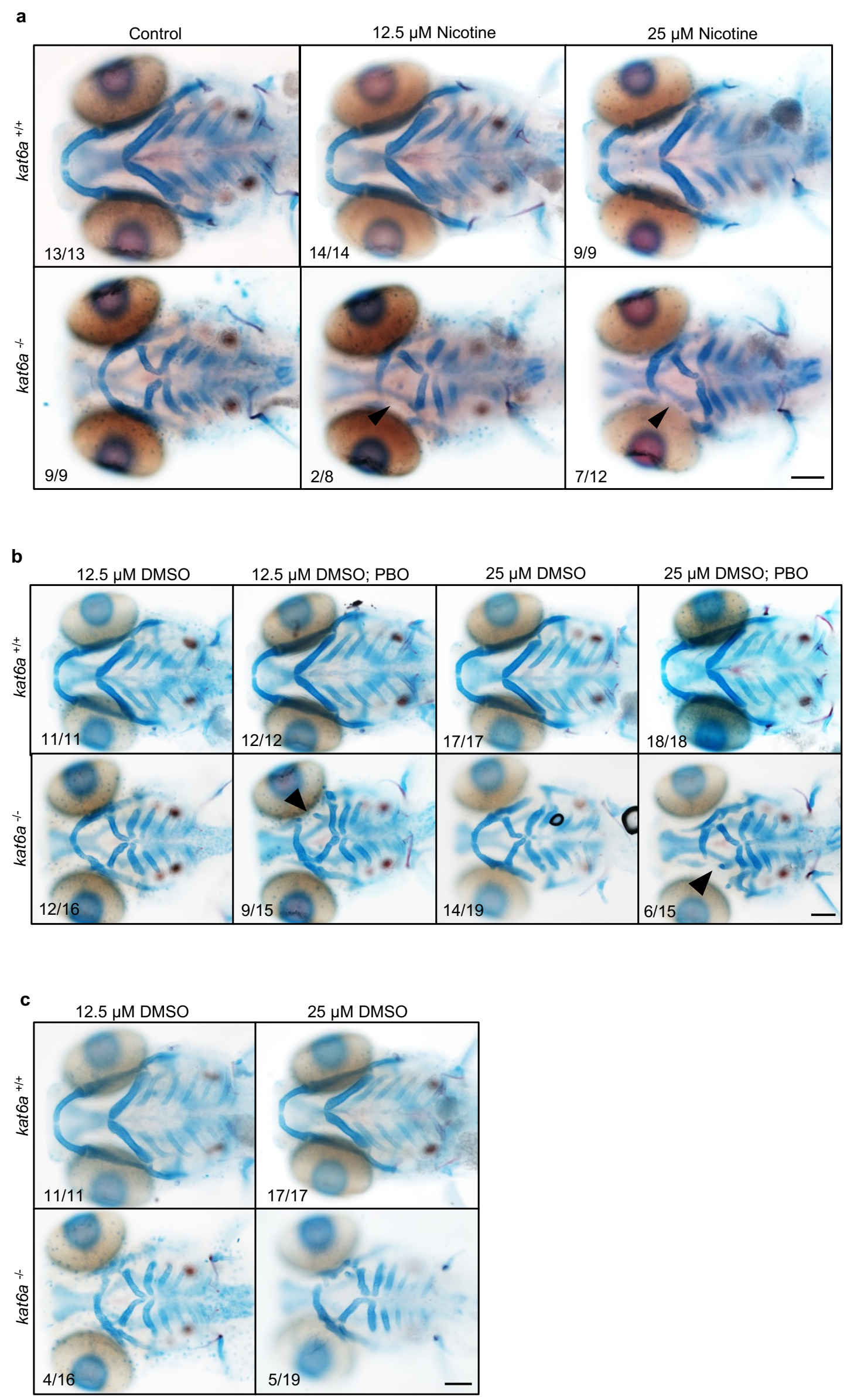

Extended Data Fig. 1| Loss of *kat6a* sensitizes embryos to a variety of environmental exposures

All images are ventral views of 4 dpf, double stained fish. **a,** Lower jaw defects in *kat6a* mutants exposed to doses of nicotine that are subteratogenic to wildtypes. Arrow heads indicate loss of Meckel's cartilage in 12.5 μM and 25 μM nicotine-exposed mutants. **b,** The common insecticide synergist and environmental contaminant PBO (piperonyl butoxide) also causes lower jaw defects in mutants (arrowheads) at doses tolerated by wildtypes. **c**, The common solvent DMSO causes defects to a fraction of exposed mutants. Scale bar = 100 μm

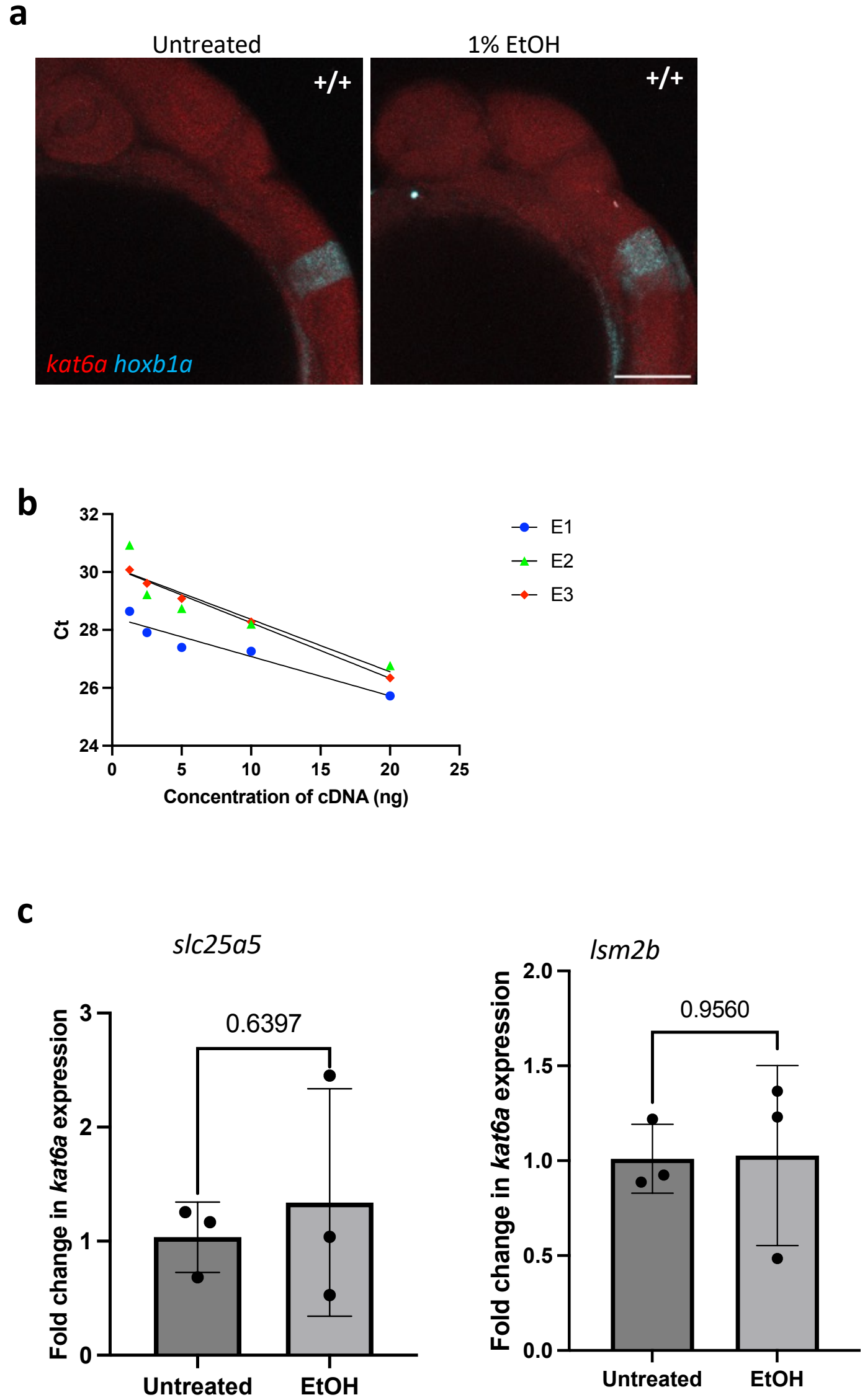

Extended Data Fig. 2| Ethanol does not alter *kat6a* transcription

a, Single-molecule fluorescent in situ hybridization at 28 hpf showing no observable differences in *kat6a* expression due to ethanol exposure. **b,** qPCR standard curve for determination of cDNA concentration and threshold cycle (Ct) for the detection of *kat6a* expression in 3 replicates (E1, E2 and E3). **c,** RT-qPCR showing relative *kat6a* expression using two different housekeeping genes (*slc25a5* and *lsm12b*) in ethanol-treated and untreated embryos at 30 hpf. Ethanol exposure did not change *kat6a* expression.

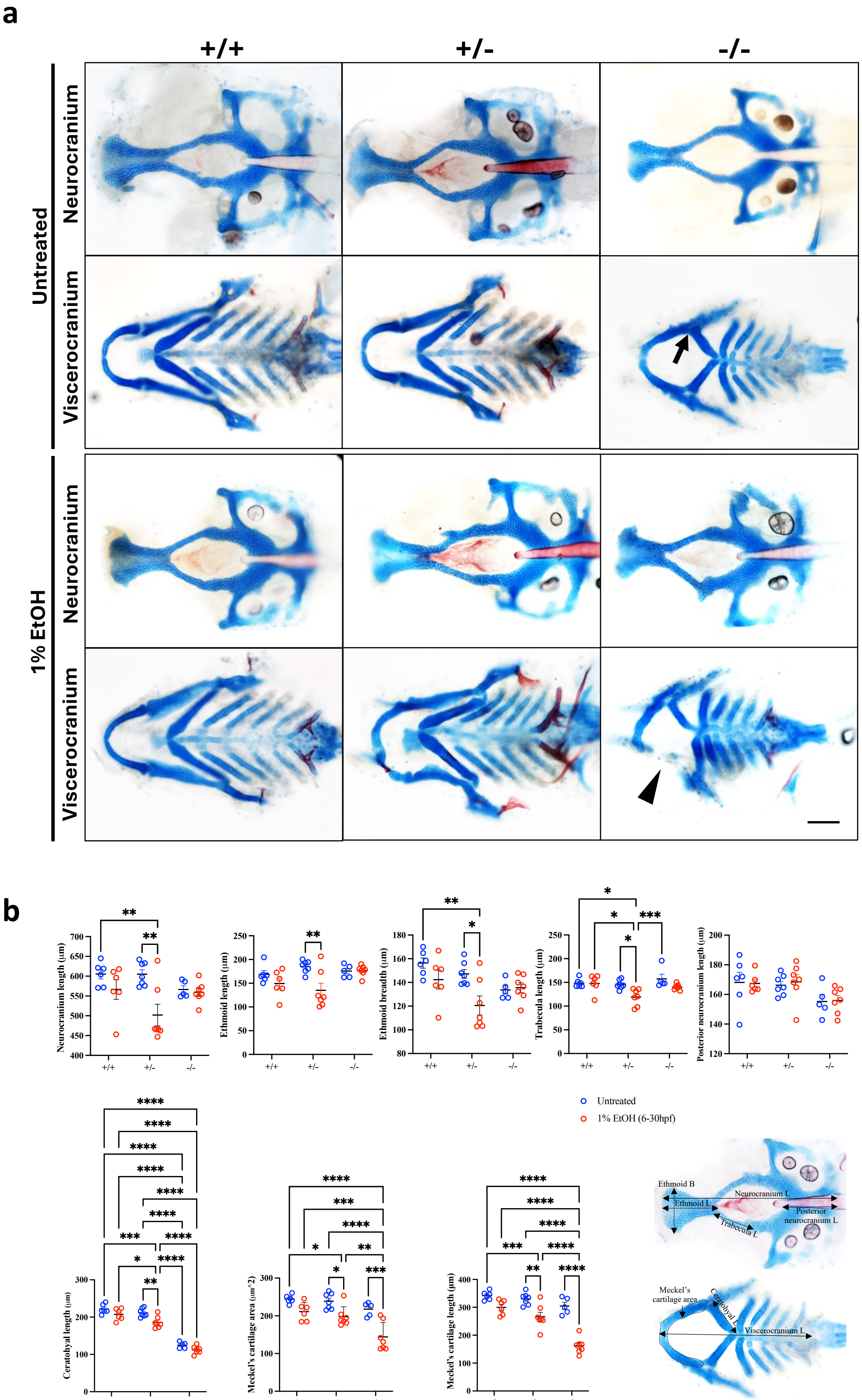

a
+/+
+/-
-/-
Untreated
1% EtOH
Neurocranium
Viscerocranium
b
Neurocranium length (μm)
Ethmoid length (μm)
Ethmoid breadth (μm)
Trabecula length (μm)
Posterior neurocranium length (μm)
Ceratohyal length (μm)
Meckel's cartilage area (μm^2)
Meckel's cartilage length (μm)
Untreated
1% EtOH (6-30hpf)
Ethmoid B
Ethmoid L
Neurocranium L
Trabecula L
Posterior neurocranium L
Meckel's cartilage area
Ceratohyal L
Viscerocranium L

Extended Data Fig. 3| Ethanol exposure particularly affects ventral mandibular skeleton in *kat6a* mutants

a, Flat mounts double stained neurocranium and viscerocranium of untreated and ethanol-treated *kat6a* wildtypes, heterozygotes and mutants. Arrow indicates posterior attachment of ceratohyal (duplicated Meckel's cartilage) on Meckel's cartilage in untreated mutants. Arrowhead indicates partial loss of Meckle's cartilage in ethanol-exposed mutant. **b,** Linear measurements of the length of the cartilaginous elements in untreated and ethanol-exposed *kat6a* wildtypes, heterozygotes and mutants. For Meckel's cartilage the area measure has also been included. Meckel's cartilage is the most profoundly disrupted skeletal element in mutants and heterozygotes. Interestingly, some measures, particularly of the neurocranium, are disrupted in ethanol-exposed heterozygotes but not mutants. * = $p < 0.05$; ** = $p < 0.01$; *** = $p < 0.001$; **** = $p < 0.0001$. Scale bar = 100 μm

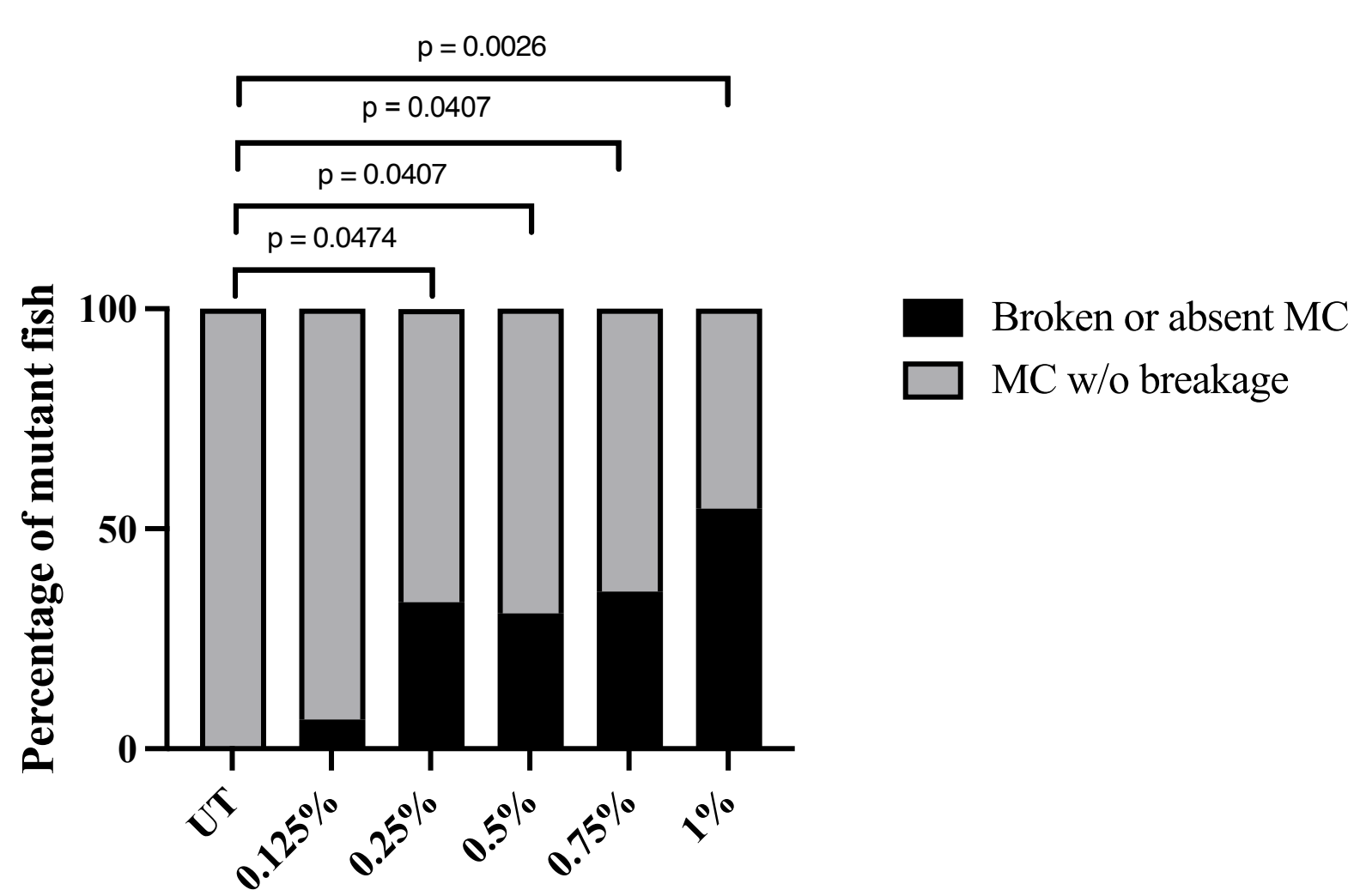

Extended Data Fig. 4| 0.25% ethanol can cause jaw defects in *kat6a* mutants

Dose response showing 0.25% 0.5%, 0.75% and 1% ethanol can induce significant jaw defects in *kat6a* mutants. Loss of Meckel's cartilage was most prevalent with 1% ethanol.

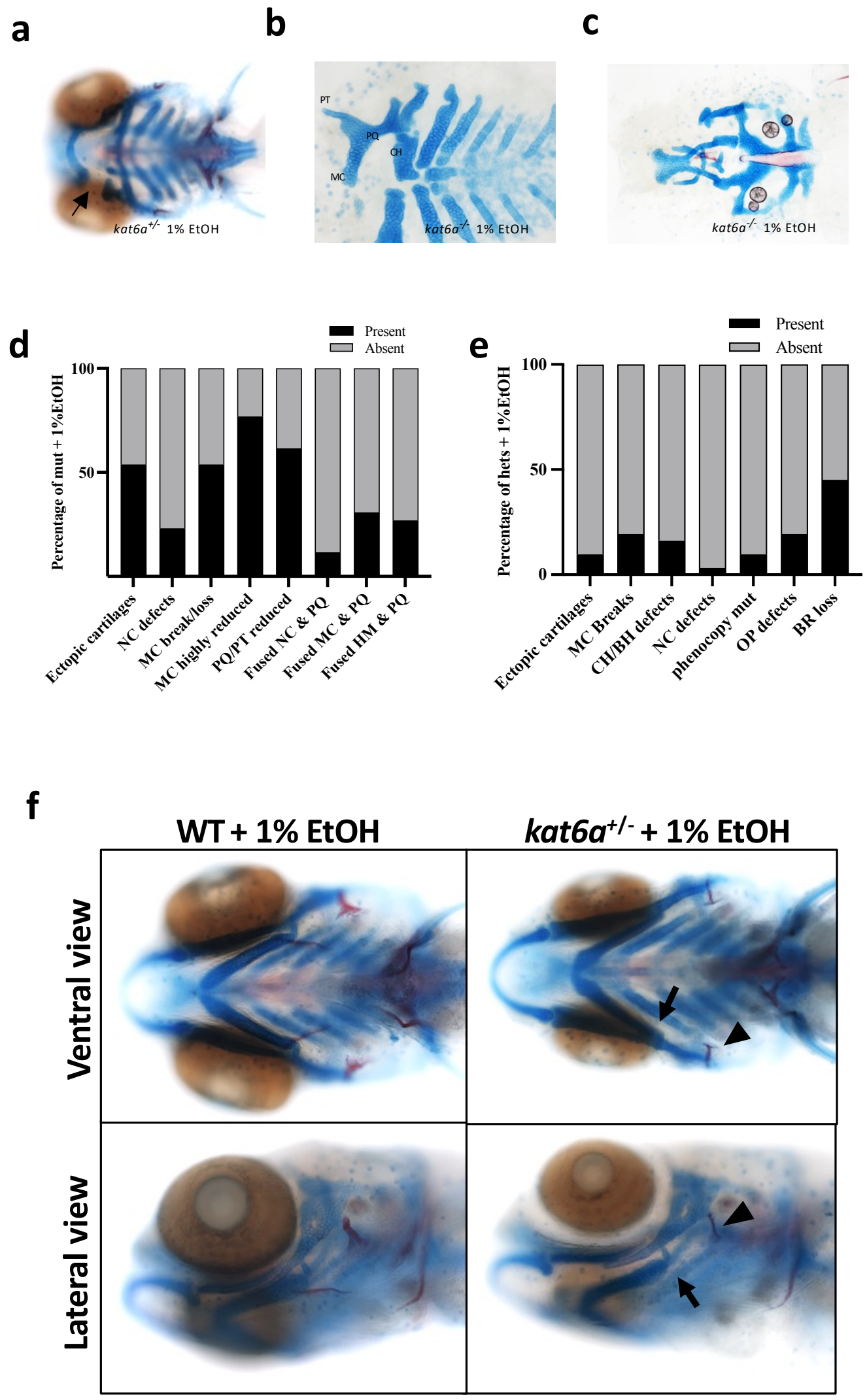

Extended Data Fig. 5| Loss of *kat6a* predisposes embryos to ethanol-induced craniofacial bone and cartilage defects

a, Whole mount of double stained, ethanol-exposed *kat6a* heterozygote showing craniofacial defects. Arrow indicates partial loss of Meckel's cartilage. **b,** Flat mount of double stained viscerocranium of ethanol-exposed *kat6a* mutant showing fusion of Meckel's cartilage and palatoquadrate. **c**, Flat mount of double stained neurocranium of ethanol-exposed *kat6a* mutant showing ectopic cartilages fused to anterior neurocranium. **d**, Percentage of *kat6a* mutants with various defects due to ethanol exposure. **e**, Percentage of *kat6a* heterozygotes with various defects due to ethanol exposure. **f**, Ethanol-exposed *kat6a* heterozygotes showing bone defects. Arrow shows loss of branchiostegal ray. Arrowhead shows opercle defects in ethanol-exposed heterozygotes. MC (Meckel's cartilage), PQ (Palatoquadrate), PT (Pterygoid process), CH (Ceratohyal), NC (Neurocranium), HM (Hyomandibula), BH (Basihyal), OP (Opercle), BR (Branchiostegal Ray)

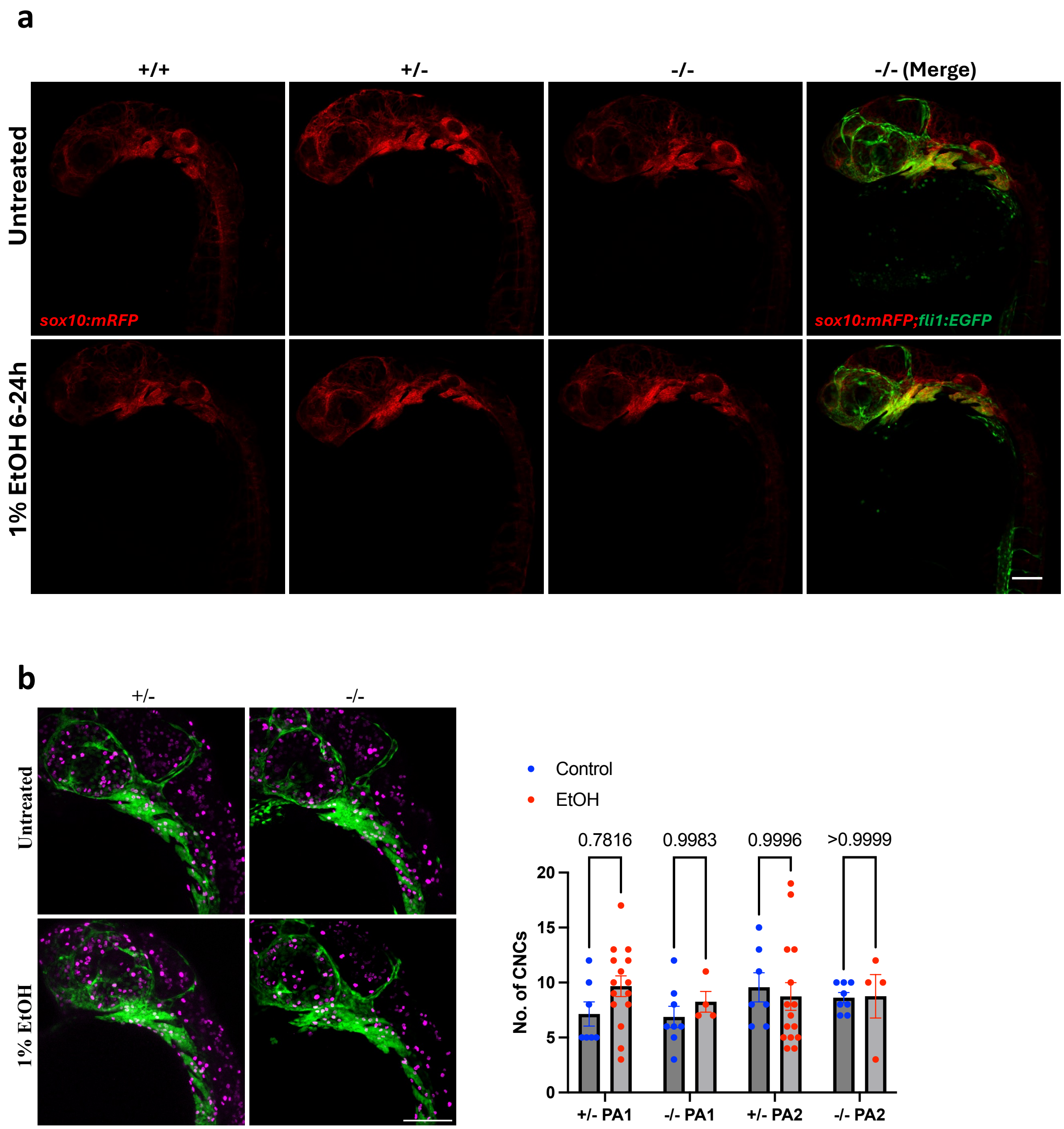

Extended Data Fig. 6| Ethanol-*kat6a* interaction does not affect gross neural crest migration or proliferation

a, Untreated and ethanol-treated *kat6a; sox10:mRFP* embryos at 28 hpf showing no defects in gross neural crest migration in the arches. **b,** Phosphohistone H3 (pHH3)-stained *kat6a* mutants and heterozygotes show no defects in neural crest cell proliferation in the arches due to ethanol exposure.

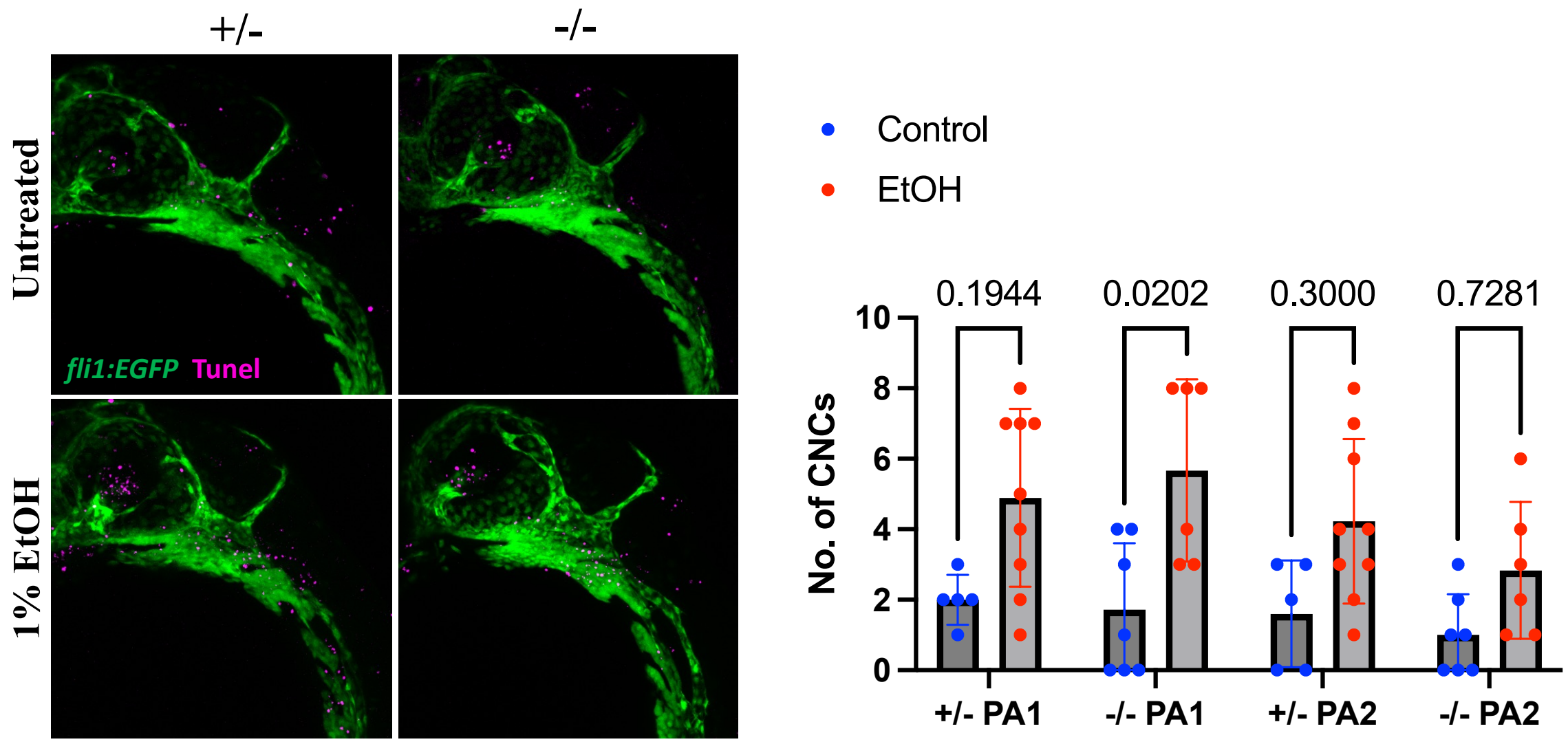

Extended Data Fig. 7| Ethanol-*kat6a* interaction elevates neural crest cell apoptosis in the mandibular arch of the mutants

Tunel staining at 28 hpf showing elevated apoptosis in the first pharyngeal arch of ethanol-exposed mutants but not heterozygotes.

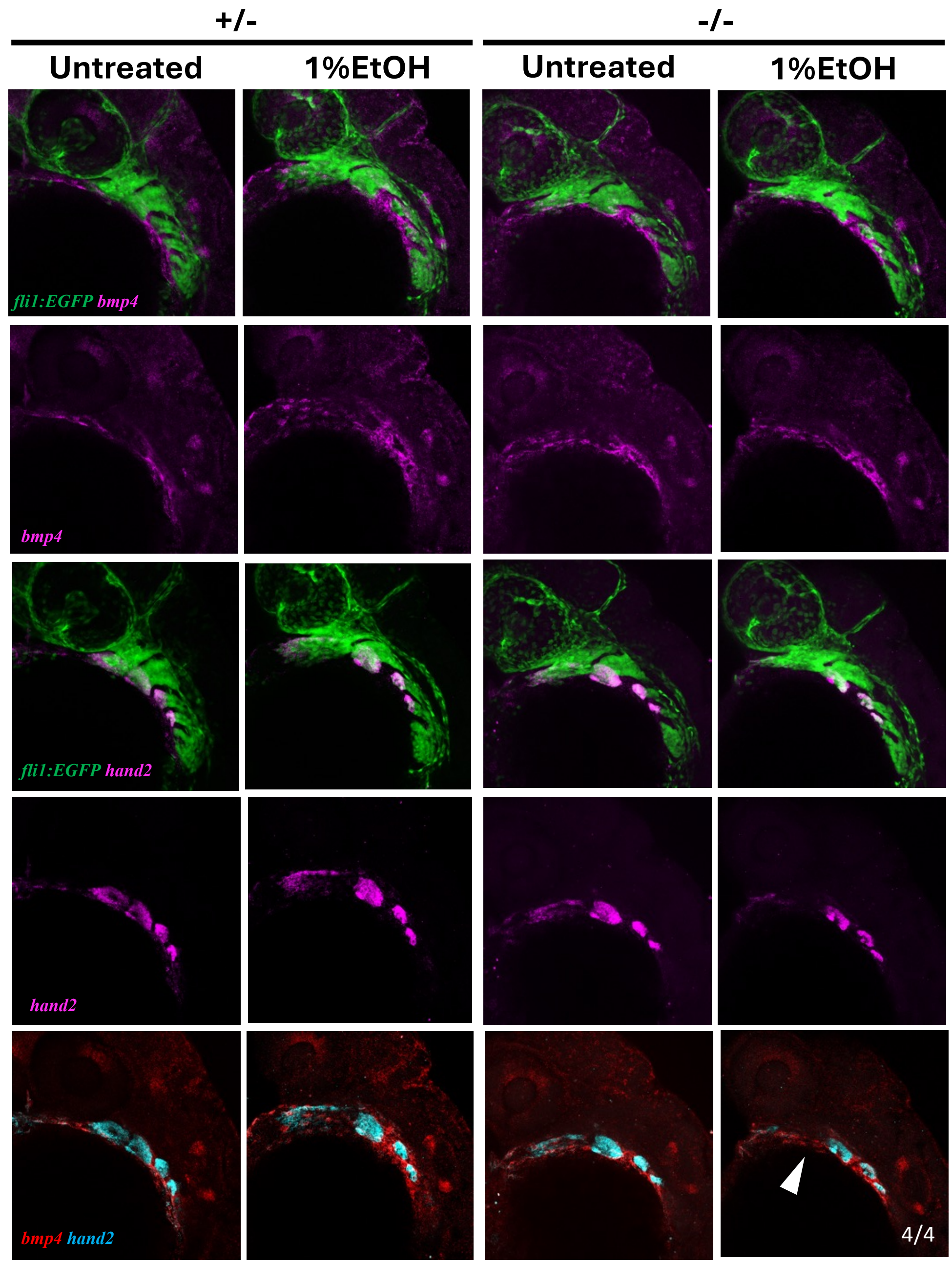

Extended Data Fig. 8| Loss of *hand2* expression is independent of *bmp4* in ethanol-exposed *kat6a* mutants

In situ hybridization of *bmp4* and *hand2* in ethanol-treated and untreated heterozygotes and mutants. The expression of *hand2*, but not *bmp4*, is lost in ethanol-exposed mutants.

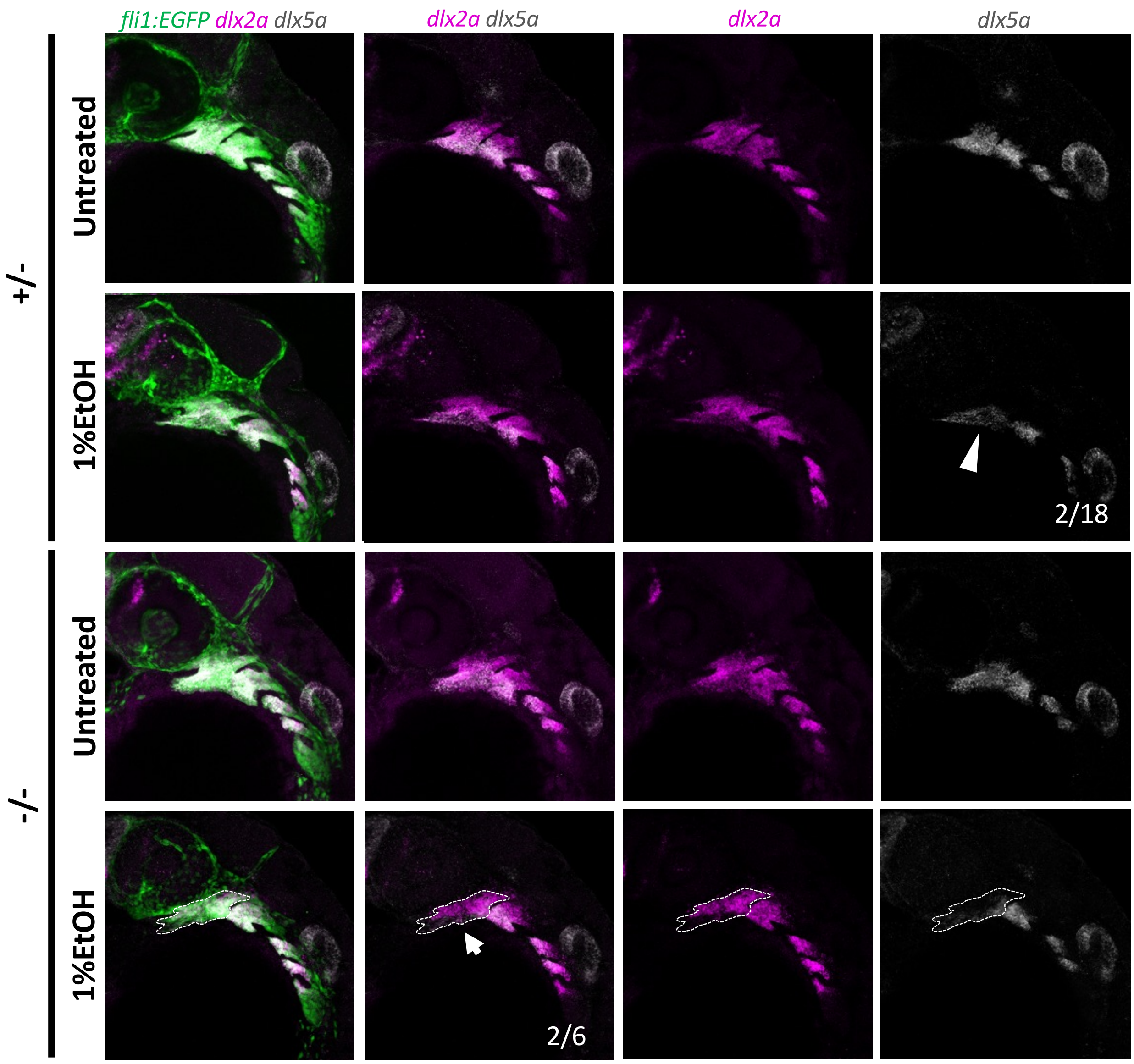

Extended Data Fig. 9| Neural crest cells lose *dlx2a* expression in the ventral mandibular arch of a subset of ethanol-exposed *kat6a* mutants

In situ hybridization of *dlx2a* and *dlx5a* in ethanol-treated and untreated heterozygotes and mutants. In a third of ethanol-exposed mutants, the expression of *dlx2a* is specifically lost in the ventral-most region of the mandibular arch (arrow). Stippled line shows the boundary of the mandibular arch in the ethanol-exposed mutant embryo. Arrowhead shows reduction in *dlx5a* in an ethanol-exposed heterozygote.

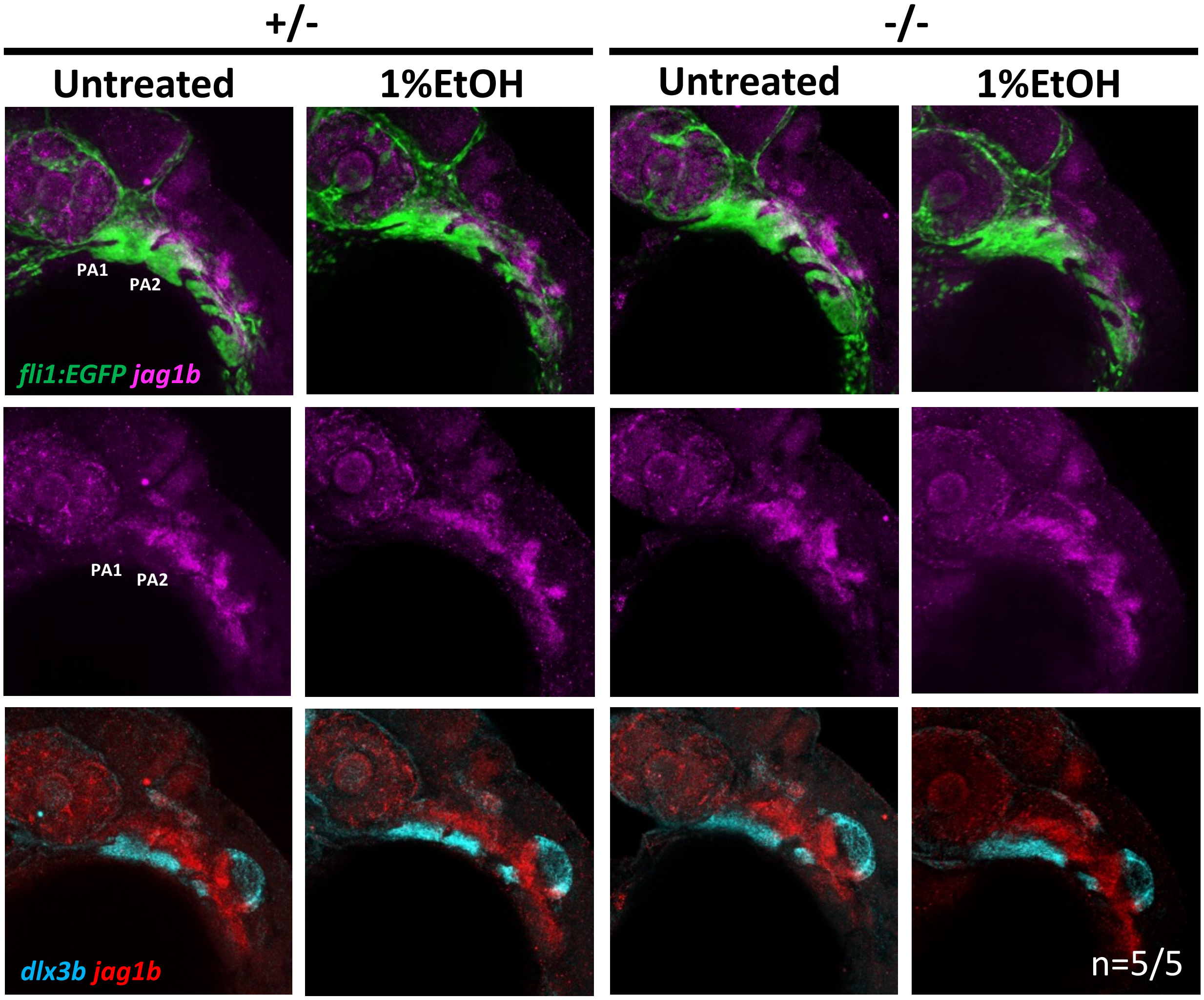

Extended Data Fig. 10| Dorsal pharyngeal arch patterning is normal in ethanol-exposed *kat6a* mutants and heterozygotes

In situ hybridization of *dlx3b* and *jag1b* in ethanol-treated and untreated heterozygotes and mutants. The expression of *jab1b* is unchanged across groups.

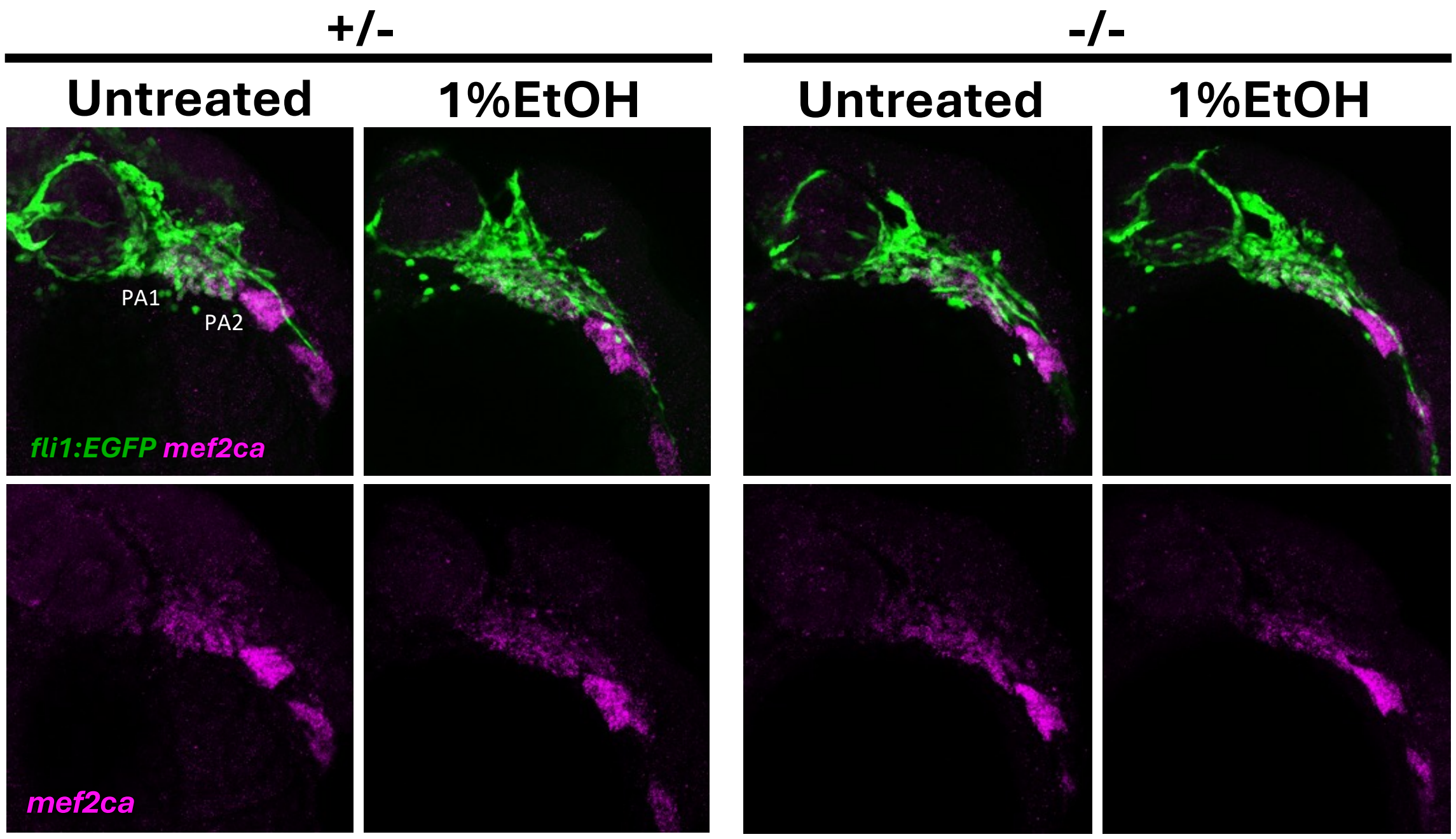

Extended Data Fig. 11| The expression of *mef2ca* is lower in the mandibular, relative to the hyoid arch.

In situ hybridization of *mef2ca* at 20 hpf. The expression of *mef2ca* is robust in the hyoid arch. Expression is lower in the mandibular arch. At this time point, ethanol exposure does not alter *mef2ca* expression in heterozygotes or mutants. PA (pharyngeal arch)

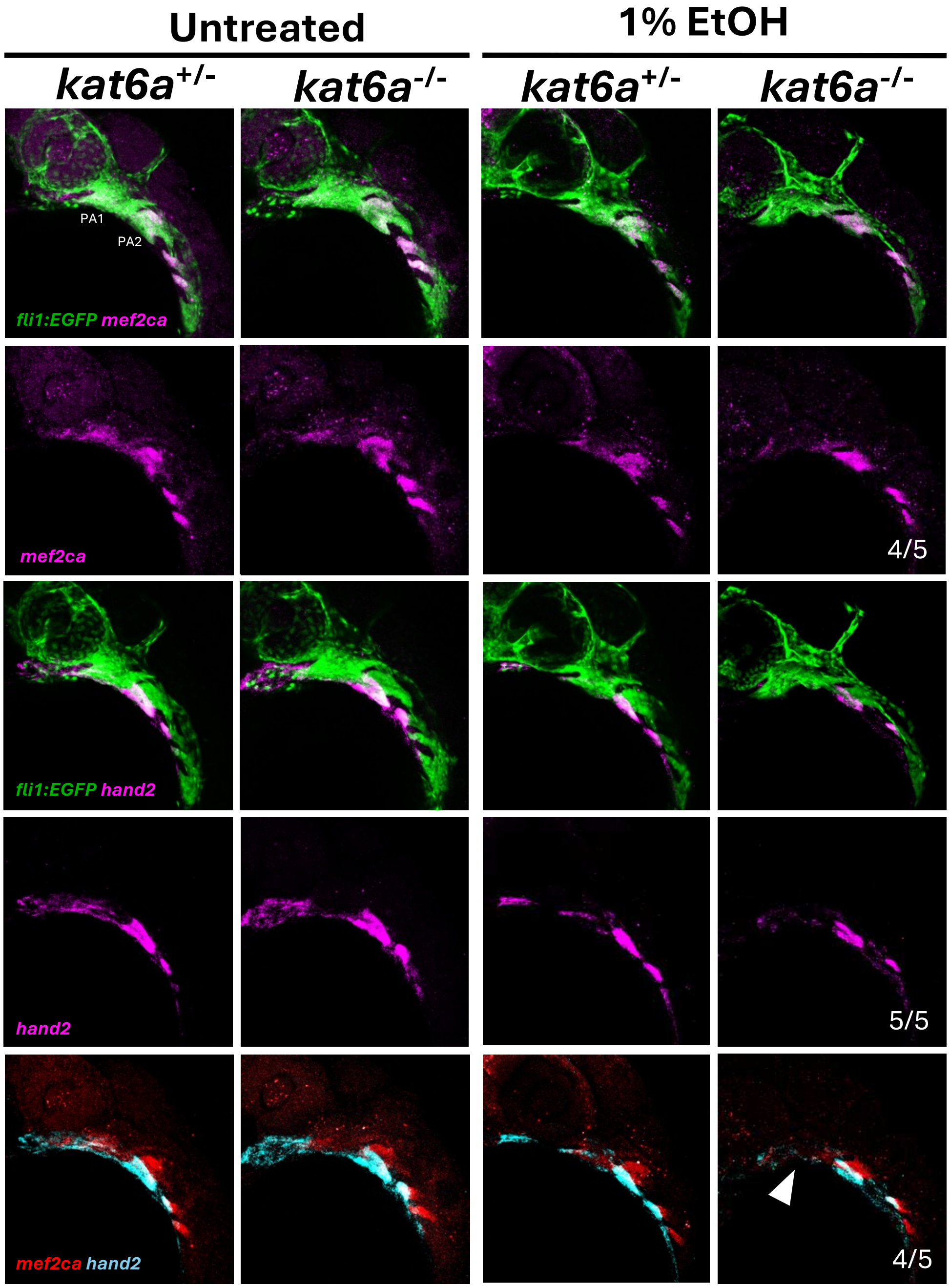

Extended Data Fig. 12| *hand2* and *mef2ca* are coordinately lost in ethanol-exposed *kat6a* mutants

In situ hybridization of *mef2ca* and *hand2* show coordinated loss of these genes in the mandibular arch of ethanol exposed mutant (indicated by the arrowhead). In regions where *hand2* is maintained, *mef2ca* is also maintained.

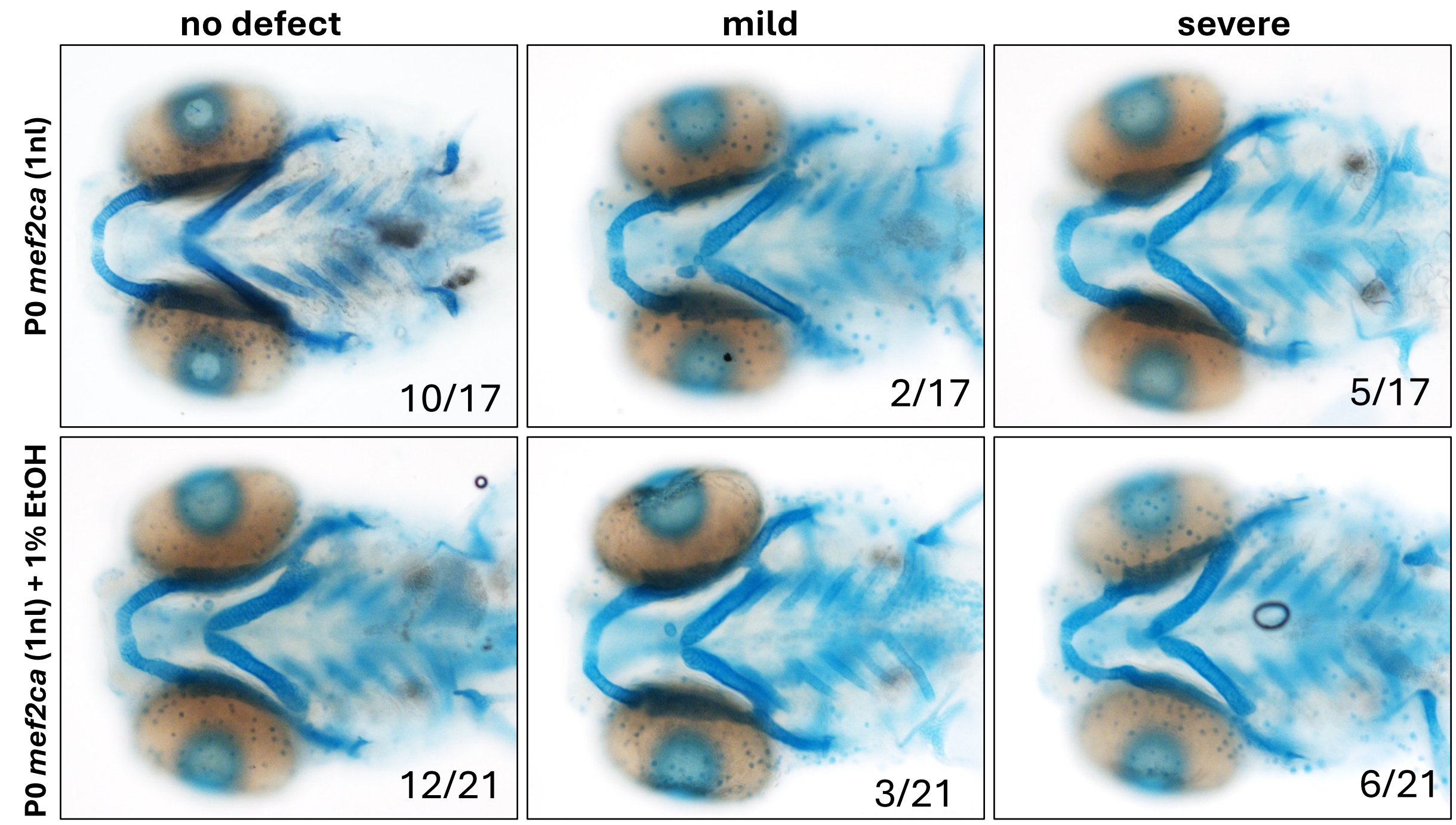

Extended Data Fig. 13| Loss of *mef2ca* does not sensitize embryos to ethanol

4 dpf, Alcian blue-stained, 1nL *mef2ca* CRISPR-injected wildtype fish show no additional defects due to ethanol exposure.

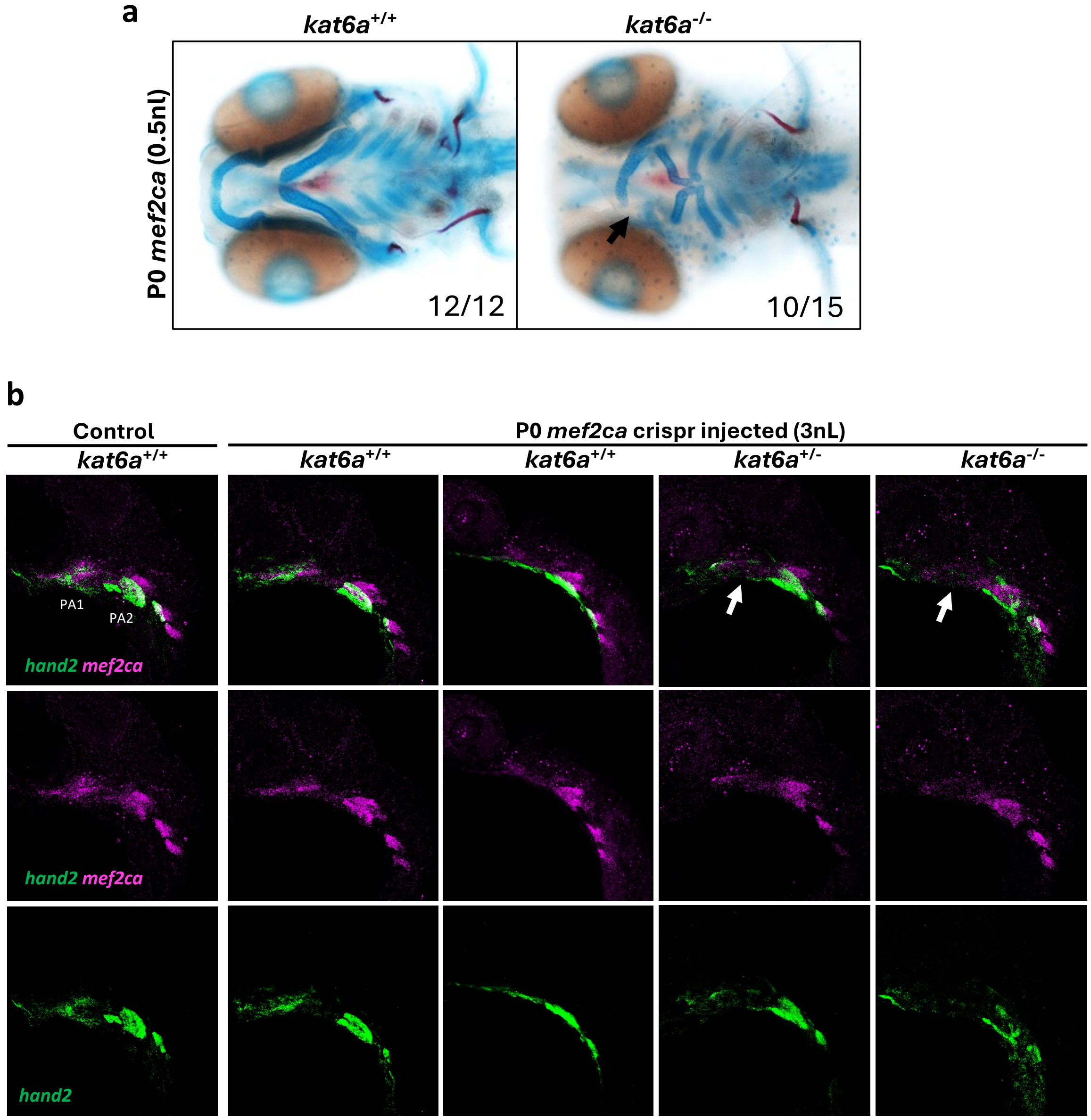

Extended Data Fig. 14| CRISPR knockdown of *mef2ca* in *kat6a* mutants downregulates *hand2* in mandibular arch and causes lower jaw breaks

a, 0.5nL *mef2ca* CRISPR-injected, double stained, *kat6a* wildtype and mutant showing loss of Meckel's cartilage in the mutant (arrow). **b**, *In situ* hybridization of *mef2ca* and *hand2* in 3nL *mef2ca* CRISPR-injected *kat6a* wildtype, heterozygote and mutant at 28 hpf. The expression of *hand2* is lost in the mandibular arch (arrows). PA (pharyngeal arch)

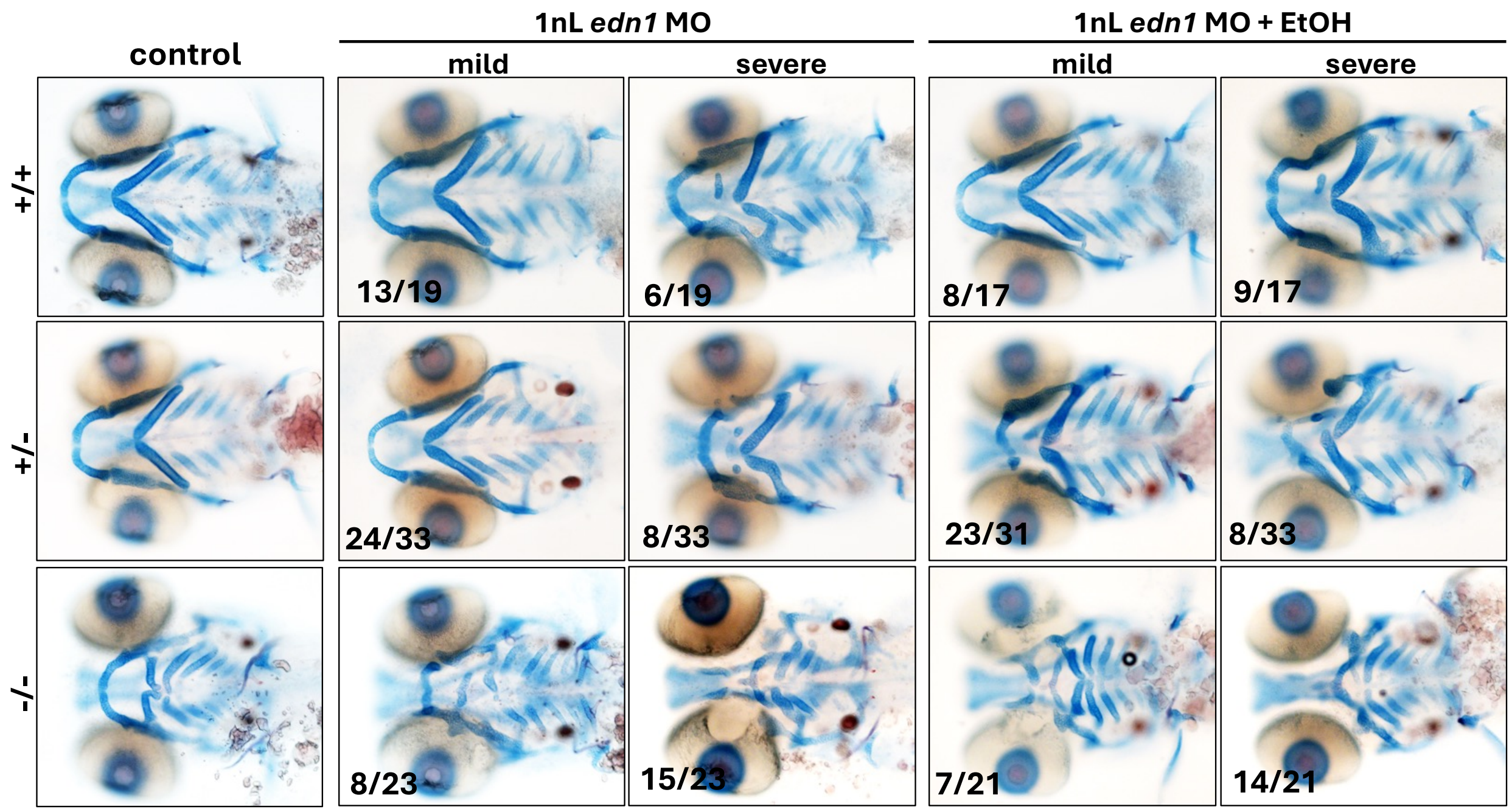

Extended Data Fig. 15I Morpholino knockdown of *edn1* exacerbate cartilage defects in *kat6a* heterozygotes and mutants

4 dpf, double stained, untreated and ethanol-treated, *edn1* morpholino-injected *kat6a* wildtypes, heterozygotes and mutants showing, loss of *edn1* exacerbates craniofacial skeletal defects in *kat6a* heterozygotes and mutants. Skeletal defects are not restricted to the mandibular arch. Loss of *edn1* does not sensitize wildtype embryos to ethanol.

Exposure window	Broken or absent MC	MC w/o breakage
Untreated	0	11
6h-24h	13	10
6h-30h	17	12
8h-24h	16	10
9h-24h	17	11
10h-24h	14	13
24h-48h	4	22
48h-4d	1	12
6h-4d	7	13

Table 1: Number of *kat6a* mutants with and without Meckel's defect in untreated and ethanol treated fish at different developmental windows. h (hours post fertilization), MC (Meckel's cartilage)

Ethanol concentration	Broken or absent MC	MC w/o breakage
Untreated	0	14
0.125%	1	14
0.25%	3	6
0.5%	4	9
0.75%	5	9
1%	6	5

Table 2: Number of *kat6a* mutants with and without Meckel's defect in untreated and fish with different ethanol concentration. MC (Meckel's cartilage)

Tukey's multiple comparisons test	Predicted (LS) mean diff.	95.00% CI of diff.	Below threshold?	Summary	Adjusted P Value
+/+:UT vs. +/+:1%EtOH	38.77	-40.15 to 117.7	No	ns	0.6740
+/+:UT vs. +/-:UT	0.2381	-75.81 to 76.29	No	ns	>0.9999
+/+:UT vs. +/-:1%EtOH	103.5	27.41 to 179.5	Yes	**	0.0031
+/+:UT vs. -/-:UT	38.79	-43.98 to 121.6	No	ns	0.7153
+/+:UT vs. -/-:1%EtOH	46.08	-29.97 to 122.1	No	ns	0.4585
+/+:1%EtOH vs. +/-:UT	-38.53	-114.6 to 37.52	No	ns	0.6453
+/+:1%EtOH vs. +/-:1%EtOH	64.69	-11.36 to 140.7	No	ns	0.1327
+/+:1%EtOH vs. -/-:UT	0.02000	-82.75 to 82.79	No	ns	>0.9999
+/+:1%EtOH vs. -/-:1%EtOH	7.314	-68.73 to 83.36	No	ns	0.9997
+/-:UT vs. +/-:1%EtOH	103.2	30.15 to 176.3	Yes	**	0.0020
+/-:UT vs. -/-:UT	38.55	-41.49 to 118.6	No	ns	0.6917
+/-:UT vs. -/-:1%EtOH	45.84	-27.22 to 118.9	No	ns	0.4202
+/-:1%EtOH vs. -/-:UT	-64.67	-144.7 to 15.37	No	ns	0.1706
+/-:1%EtOH vs. -/-:1%EtOH	-57.37	-130.4 to 15.69	No	ns	0.1940
-/-:UT vs. -/-:1%EtOH	7.294	-72.74 to 87.33	No	ns	0.9998

Table 3: Two-way ANOVA with Tukey's multiple comparisons correction to analyze if ethanol affects the length of neurocranium in *kat6a* mutants and heterozygotes.

Tukey's multiple comparisons test	Predicted (LS) mean diff.	95.00% CI of diff.	Below threshold?	Summary	Adjusted P Value
+/+:UT vs. +/+:1% EtOH	19.52	-21.42 to 60.45	No	ns	0.7005
+/+:UT vs. +/-:UT	-16.23	-55.67 to 23.22	No	ns	0.8109
+/+:UT vs. +/-:1% EtOH	33.73	-5.712 to 73.17	No	ns	0.1290
+/+:UT vs. -/-:UT	-6.723	-49.65 to 36.21	No	ns	0.9967
+/+:UT vs. -/-:1% EtOH	-7.655	-47.10 to 31.79	No	ns	0.9911
+/+:1% EtOH vs. +/-:UT	-35.74	-75.19 to 3.700	No	ns	0.0938
+/+:1% EtOH vs. +/-:1% EtOH	14.21	-25.23 to 53.66	No	ns	0.8811
+/+:1% EtOH vs. -/-:UT	-26.24	-69.17 to 16.69	No	ns	0.4490
+/+:1% EtOH vs. -/-:1% EtOH	-27.17	-66.61 to 12.27	No	ns	0.3194
+/-:UT vs. +/-:1% EtOH	49.96	12.06 to 87.85	Yes	**	0.0044
+/-:UT vs. -/-:UT	9.503	-32.01 to 51.02	No	ns	0.9814
+/-:UT vs. -/-:1% EtOH	8.571	-29.32 to 46.47	No	ns	0.9824
+/-:1% EtOH vs. -/-:UT	-40.45	-81.97 to 1.059	No	ns	0.0596
+/-:1% EtOH vs. -/-:1% EtOH	-41.39	-79.28 to -3.490	Yes	*	0.0258
-/-:UT vs. -/-:1% EtOH	-0.9314	-42.44 to 40.58	No	ns	>0.9999

Table 4: Two-way ANOVA with Tukey's multiple comparisons correction to analyze if ethanol affects the ethmoid length in *kat6a* mutants and heterozygotes.

Tukey's multiple comparisons test	Predicted (LS) mean diff.	95.00% CI of diff.	Below threshold?	Summary	Adjusted P Value
+/+:UT vs. +/+:1% EtOH	19.52	-21.42 to 60.45	No	ns	0.7005
+/+:UT vs. +/-:UT	-16.23	-55.67 to 23.22	No	ns	0.8109
+/+:UT vs. +/-:1% EtOH	33.73	-5.712 to 73.17	No	ns	0.1290
+/+:UT vs. -/-:UT	-6.723	-49.65 to 36.21	No	ns	0.9967
+/+:UT vs. -/-:1% EtOH	-7.655	-47.10 to 31.79	No	ns	0.9911
+/+:1% EtOH vs. +/-:UT	-35.74	-75.19 to 3.700	No	ns	0.0938
+/+:1% EtOH vs. +/-:1% EtOH	14.21	-25.23 to 53.66	No	ns	0.8811
+/+:1% EtOH vs. -/-:UT	-26.24	-69.17 to 16.69	No	ns	0.4490
+/+:1% EtOH vs. -/-:1% EtOH	-27.17	-66.61 to 12.27	No	ns	0.3194
+/-:UT vs. +/-:1% EtOH	49.96	12.06 to 87.85	Yes	**	0.0044
+/-:UT vs. -/-:UT	9.503	-32.01 to 51.02	No	ns	0.9814
+/-:UT vs. -/-:1% EtOH	8.571	-29.32 to 46.47	No	ns	0.9824
+/-:1% EtOH vs. -/-:UT	-40.45	-81.97 to 1.059	No	ns	0.0596
+/-:1% EtOH vs. -/-:1% EtOH	-41.39	-79.28 to -3.490	Yes	*	0.0258
-/-:UT vs. -/-:1% EtOH	-0.9314	-42.44 to 40.58	No	ns	>0.9999

Table 5: Two-way ANOVA with Tukey's multiple comparisons correction to analyze if ethanol affects the ethmoid breadth in *kat6a* mutants and heterozygotes.

Tukey's multiple comparisons test	Predicted (LS) mean diff.	95.00% CI of diff.	Below threshold?	Summary	Adjusted P Value
+/+:UT vs. +/+:1% EtOH	19.52	-21.42 to 60.45	No	ns	0.7005
+/+:UT vs. +/-:UT	-16.23	-55.67 to 23.22	No	ns	0.8109
+/+:UT vs. +/-:1% EtOH	33.73	-5.712 to 73.17	No	ns	0.1290
+/+:UT vs. -/-:UT	-6.723	-49.65 to 36.21	No	ns	0.9967
+/+:UT vs. -/-:1% EtOH	-7.655	-47.10 to 31.79	No	ns	0.9911
+/+:1% EtOH vs. +/-:UT	-35.74	-75.19 to 3.700	No	ns	0.0938
+/+:1% EtOH vs. +/-:1% EtOH	14.21	-25.23 to 53.66	No	ns	0.8811
+/+:1% EtOH vs. -/-:UT	-26.24	-69.17 to 16.69	No	ns	0.4490
+/+:1% EtOH vs. -/-:1% EtOH	-27.17	-66.61 to 12.27	No	ns	0.3194
+/-:UT vs. +/-:1% EtOH	49.96	12.06 to 87.85	Yes	**	0.0044
+/-:UT vs. -/-:UT	9.503	-32.01 to 51.02	No	ns	0.9814
+/-:UT vs. -/-:1% EtOH	8.571	-29.32 to 46.47	No	ns	0.9824
+/-:1% EtOH vs. -/-:UT	-40.45	-81.97 to 1.059	No	ns	0.0596
+/-:1% EtOH vs. -/-:1% EtOH	-41.39	-79.28 to -3.490	Yes	*	0.0258
-/-:UT vs. -/-:1% EtOH	-0.9314	-42.44 to 40.58	No	ns	>0.9999

Table 6: Two-way ANOVA with Tukey's multiple comparisons correction to analyze if ethanol affects the length of trabecula in *kat6a* mutants and heterozygotes.

Tukey's multiple comparisons test	Predicted (LS) mean diff.	95.00% CI of diff.	Below threshold?	Summary	Adjusted P Value
+/+:UT vs. +/+:1% EtOH	0.6833	-18.23 to 19.60	No	ns	>0.9999
+/+:UT vs. +/-:UT	1.843	-16.38 to 20.07	No	ns	0.9996
+/+:UT vs. +/-:1% EtOH	-0.3429	-18.57 to 17.88	No	ns	>0.9999
+/+:UT vs. -/-:UTc	12.84	-6.998 to 32.68	No	ns	0.3861
+/+:UT vs. -/-:1% EtOH	12.21	-6.012 to 30.44	No	ns	0.3486
+/+:1% EtOH vs. +/-:UT	1.160	-17.07 to 19.39	No	ns	>0.9999
+/+:1% EtOH vs. +/-:1% EtOH	-1.026	-19.25 to 17.20	No	ns	>0.9999
+/+:1% EtOH vs. -/-:UT	12.16	-7.681 to 31.99	No	ns	0.4462
+/+:1% EtOH vs. -/-:1% EtOH	11.53	-6.696 to 29.76	No	ns	0.4111
+/-:UT vs. +/-:1% EtOH	-2.186	-19.70 to 15.33	No	ns	0.9989
+/-:UT vs. -/-:UT	11.00	-8.186 to 30.18	No	ns	0.5190
+/-:UT vs. -/-:1% EtOH	10.37	-7.140 to 27.88	No	ns	0.4836
+/-:1% EtOH vs. -/-:UT	13.18	-6.000 to 32.37	No	ns	0.3219
+/-:1% EtOH vs. -/-:1% EtOH	12.56	-4.955 to 30.07	No	ns	0.2783
-/-:UT vs. -/-:1% EtOH	-0.6257	-19.81 to 18.56	No	ns	>0.9999

Table 7: Two-way ANOVA with Tukey's multiple comparisons correction to analyze if ethanol affects the length of posterior neurocranium in *kat6a* mutants and heterozygotes.

Tukey's multiple comparisons test	Predicted (LS) mean diff.	95.00% CI of diff.	Below threshold?	Summary	Adjusted P Value
+/+:UT vs. +/+:1% EtOH	14.23	-7.773 to 36.24	No	ns	0.3869
+/+:UT vs. +/-:UT	9.989	-11.22 to 31.19	No	ns	0.7108
+/+:UT vs. +/-:1% EtOH	35.53	14.33 to 56.74	Yes	***	0.0002
+/+:UT vs. -/-:UT	96.69	73.60 to 119.8	Yes	****	<0.0001
+/+:UT vs. -/-:1% EtOH	109.3	88.08 to 130.5	Yes	****	<0.0001
+/+:1% EtOH vs. +/-:UT	-4.244	-25.45 to 16.96	No	ns	0.9898
+/+:1% EtOH vs. +/-:1% EtOH	21.30	0.09317 to 42.50	Yes	*	0.0485
+/+:1% EtOH vs. -/-:UT	82.45	59.37 to 105.5	Yes	****	<0.0001
+/+:1% EtOH vs. -/-:1% EtOH	95.05	73.84 to 116.3	Yes	****	<0.0001
+/-:UT vs. +/-:1% EtOH	25.54	5.169 to 45.92	Yes	**	0.0074
+/-:UT vs. -/-:UT	86.70	64.38 to 109.0	Yes	****	<0.0001
+/-:UT vs. -/-:1% EtOH	99.29	78.92 to 119.7	Yes	****	<0.0001
+/-:1% EtOH vs. -/-:UT	61.15	38.83 to 83.47	Yes	****	<0.0001
+/-:1% EtOH vs. -/-:1% EtOH	73.75	53.38 to 94.12	Yes	****	<0.0001
-/-:UT vs. -/-:1% EtOH	12.60	-9.721 to 34.92	No	ns	0.5356

Table 8: Two-way ANOVA with Tukey's multiple comparisons correction to analyze if ethanol affects the ceratohyal length in *kat6a* mutants and heterozygotes.

Tukey's multiple comparisons test	Predicted (LS) mean diff.	95.00% CI of diff.	Below threshold?	Summary	Adjusted P Value
+/+:UT vs. +/+:1%	33.31	-10.79 to 77.41	No	ns	0.2284
+/+:UT vs. +/-:UT	5.748	-36.75 to 48.24	No	ns	0.9984
+/+:UT vs. +/-:1%	46.93	4.437 to 89.43	Yes	*	0.0235
+/+:UT vs. -/-:UT	26.75	-19.50 to 73.01	No	ns	0.5093
+/+:UT vs. -/-:1%	100.2	57.72 to 142.7	Yes	****	<0.0001
+/+:1% vs. +/-:UT	-27.56	-70.06 to 14.93	No	ns	0.3839
+/+:1% vs. +/-:1%	13.62	-28.87 to 56.12	No	ns	0.9237
+/+:1% vs. -/-:UT	-6.557	-52.81 to 39.70	No	ns	0.9980
+/+:1% vs. -/-:1%	66.91	24.41 to 109.4	Yes	***	0.0005
+/-:UT vs. +/-:1%	41.19	0.3564 to 82.01	Yes	*	0.0470
+/-:UT vs. -/-:UT	21.01	-23.72 to 65.73	No	ns	0.7134
+/-:UT vs. -/-:1%	94.47	53.64 to 135.3	Yes	****	<0.0001
+/-:1% vs. -/-:UT	-20.18	-64.91 to 24.55	No	ns	0.7459
+/-:1% vs. -/-:1%	53.29	12.46 to 94.11	Yes	**	0.0049
-/-:UT vs. -/-:1%	73.47	28.74 to 118.2	Yes	***	0.0003

Table 9: Two-way ANOVA with Tukey's multiple comparisons correction to analyze if ethanol affects the area of the Meckel's cartilage in *kat6a* mutants and heterozygotes.

Tukey's multiple comparisons test	Predicted (LS) Mean diff.	95.00% CI of diff.	Below threshold?	Summary	Adjusted P Value
+/+:UT vs. +/+:1%	42.17	-8.222 to 92.56	No	ns	0.1441
+/+:UT vs. +/-:UT	11.89	-36.66 to 60.45	No	ns	0.9750
+/+:UT vs. +/-:1%	75.61	27.05 to 124.2	Yes	***	0.0006
+/+:UT vs. -/-:UT	37.09	-15.76 to 89.94	No	ns	0.3001
+/+:UT vs. -/-:1%	179.8	131.2 to 228.3	Yes	****	<0.0001
+/+:1% vs. +/-:UT	-30.27	-78.83 to 18.28	No	ns	0.4271
+/+:1% vs. +/-:1%	33.44	-15.12 to 82.00	No	ns	0.3196
+/+:1% vs. -/-:UT	-5.077	-57.93 to 47.77	No	ns	0.9997
+/+:1% vs. -/-:1%	137.6	89.07 to 186.2	Yes	****	<0.0001
+/-:UT vs. +/-:1%	63.71	17.06 to 110.4	Yes	**	0.0030
+/-:UT vs. -/-:UT	25.20	-25.91 to 76.30	No	ns	0.6707
+/-:UT vs. -/-:1%	167.9	121.2 to 214.6	Yes	****	<0.0001
+/-:1% vs. -/-:UT	-38.52	-89.62 to 12.59	No	ns	0.2304
+/-:1% vs. -/-:1%	104.2	57.53 to 150.8	Yes	****	<0.0001
-/-:UT vs. -/-:1%	142.7	91.60 to 193.8	Yes	****	<0.0001

Table 10: Two-way ANOVA with Tukey's multiple comparisons correction to analyze if ethanol affects the length of the Meckel's cartilage in *kat6a* mutants and heterozygotes.

Tukey's multiple comparisons test	Predicted (LS) mean diff.	95.00% CI of diff.	Below threshold?	Summary	Adjusted P Value
+/- PA1:Control vs. +/- PA1:EtOH	-2.889	-6.461 to 0.6833	No	ns	0.1944
+/- PA1:Control vs. -/- PA1:Control	0.2857	-3.464 to 4.036	No	ns	>0.9999
+/- PA1:Control vs. -/- PA1:EtOH	-3.667	-7.545 to 0.2114	No	ns	0.0760
+/- PA1:Control vs. +/- PA2:Control	0.4000	-3.651 to 4.451	No	ns	>0.9999
+/- PA1:Control vs. +/- PA2:EtOH	-2.222	-5.794 to 1.350	No	ns	0.5093
+/- PA1:Control vs. -/- PA2:Control	1.000	-2.750 to 4.750	No	ns	0.9892
+/- PA1:Control vs. -/- PA2:EtOH	-0.8333	-4.711 to 3.045	No	ns	0.9971
+/- PA1:EtOH vs. -/- PA1:Control	3.175	-0.05293 to 6.402	No	ns	0.0569
+/- PA1:EtOH vs. -/- PA1:EtOH	-0.7778	-4.153 to 2.598	No	ns	0.9955
+/- PA1:EtOH vs. +/- PA2:Control	3.289	-0.2833 to 6.861	No	ns	0.0913
+/- PA1:EtOH vs. +/- PA2:EtOH	0.6667	-2.352 to 3.686	No	ns	0.9966
+/- PA1:EtOH vs. -/- PA2:Control	3.889	0.6614 to 7.116	Yes	**	0.0086
+/- PA1:EtOH vs. -/- PA2:EtOH	2.056	-1.320 to 5.431	No	ns	0.5363
-/- PA1:Control vs. -/- PA1:EtOH	-3.952	-7.515 to -0.3893	Yes	*	0.0202
-/- PA1:Control vs. +/- PA2:Control	0.1143	-3.636 to 3.864	No	ns	>0.9999
-/- PA1:Control vs. +/- PA2:EtOH	-2.508	-5.735 to 0.7196	No	ns	0.2355
-/- PA1:Control vs. -/- PA2:Control	0.7143	-2.709 to 4.138	No	ns	0.9976
-/- PA1:Control vs. -/- PA2:EtOH	-1.119	-4.682 to 2.444	No	ns	0.9726
-/- PA1:EtOH vs. +/- PA2:Control	4.067	0.1886 to 7.945	Yes	*	0.0338
-/- PA1:EtOH vs. +/- PA2:EtOH	1.444	-1.931 to 4.820	No	ns	0.8709
-/- PA1:EtOH vs. -/- PA2:Control	4.667	1.104 to 8.230	Yes	**	0.0032
-/- PA1:EtOH vs. -/- PA2:EtOH	2.833	-0.8643 to 6.531	No	ns	0.2507
+/- PA2:Control vs. +/- PA2:EtOH	-2.622	-6.194 to 0.9500	No	ns	0.3000
+/- PA2:Control vs. -/- PA2:Control	0.6000	-3.150 to 4.350	No	ns	0.9996
+/- PA2:Control vs. -/- PA2:EtOH	-1.233	-5.111 to 2.645	No	ns	0.9707
+/- PA2:EtOH vs. -/- PA2:Control	3.222	-0.005314 to 6.450	No	ns	0.0507
+/- PA2:EtOH vs. -/- PA2:EtOH	1.389	-1.987 to 4.764	No	ns	0.8919
-/- PA2:Control vs. -/- PA2:EtOH	-1.833	-5.396 to 1.730	No	ns	0.7281

Table 11: Two-way ANOVA with Tukey’s multiple comparisons correction was used to examine if ethanol elevated neural crest cell death in pharyngeal arch (PA)1 and PA2 in *kat6a* mutants and heterozygotes.

Tukey's multiple comparisons test	Predicted (LS) mean diff.	95.00% CI of diff.	Below threshold?	Summary	Adjusted P Value
+/- PA1:Control vs. +/- PA1:EtOH	-2.524	-7.664 to 2.616	No	ns	0.7816
+/- PA1:Control vs. -/- PA1:Control	0.2679	-5.543 to 6.079	No	ns	>0.9999
+/- PA1:Control vs. -/- PA1:EtOH	-1.107	-8.145 to 5.931	No	ns	0.9997
+/- PA1:Control vs. +/- PA2:Control	-2.429	-8.430 to 3.573	No	ns	0.9060
+/- PA1:Control vs. +/- PA2:EtOH	-1.590	-6.730 to 3.549	No	ns	0.9767
+/- PA1:Control vs. -/- PA2:Control	-1.482	-7.293 to 4.329	No	ns	0.9924
+/- PA1:Control vs. -/- PA2:EtOH	-1.607	-8.645 to 5.431	No	ns	0.9962
+/- PA1:EtOH vs. -/- PA1:Control	2.792	-2.124 to 7.707	No	ns	0.6334
+/- PA1:EtOH vs. -/- PA1:EtOH	1.417	-4.902 to 7.735	No	ns	0.9966
+/- PA1:EtOH vs. +/- PA2:Control	0.09524	-5.044 to 5.235	No	ns	>0.9999
+/- PA1:EtOH vs. +/- PA2:EtOH	0.9333	-3.167 to 5.033	No	ns	0.9962
+/- PA1:EtOH vs. -/- PA2:Control	1.042	-3.874 to 5.957	No	ns	0.9976
+/- PA1:EtOH vs. -/- PA2:EtOH	0.9167	-5.402 to 7.235	No	ns	0.9998
-/- PA1:Control vs. -/- PA1:EtOH	-1.375	-8.251 to 5.501	No	ns	0.9983
-/- PA1:Control vs. +/- PA2:Control	-2.696	-8.508 to 3.115	No	ns	0.8264
-/- PA1:Control vs. +/- PA2:EtOH	-1.858	-6.774 to 3.057	No	ns	0.9325
-/- PA1:Control vs. -/- PA2:Control	-1.750	-7.364 to 3.864	No	ns	0.9758
-/- PA1:Control vs. -/- PA2:EtOH	-1.875	-8.751 to 5.001	No	ns	0.9887
-/- PA1:EtOH vs. +/- PA2:Control	-1.321	-8.359 to 5.716	No	ns	0.9989
-/- PA1:EtOH vs. +/- PA2:EtOH	-0.4833	-6.802 to 5.835	No	ns	>0.9999
-/- PA1:EtOH vs. -/- PA2:Control	-0.3750	-7.251 to 6.501	No	ns	>0.9999
-/- PA1:EtOH vs. -/- PA2:EtOH	-0.5000	-8.440 to 7.440	No	ns	>0.9999
+/- PA2:Control vs. +/- PA2:EtOH	0.8381	-4.302 to 5.978	No	ns	0.9996
+/- PA2:Control vs. -/- PA2:Control	0.9464	-4.865 to 6.758	No	ns	0.9996
+/- PA2:Control vs. -/- PA2:EtOH	0.8214	-6.216 to 7.859	No	ns	>0.9999
+/- PA2:EtOH vs. -/- PA2:Control	0.1083	-4.807 to 5.024	No	ns	>0.9999
+/- PA2:EtOH vs. -/- PA2:EtOH	-0.01667	-6.335 to 6.302	No	ns	>0.9999
-/- PA2:Control vs. -/- PA2:EtOH	-0.1250	-7.001 to 6.751	No	ns	>0.9999

Table 12: Two-way ANOVA with Tukey's multiple comparisons correction was used to examine if ethanol affects neural crest cell proliferation in pharyngeal arch (PA)1 and PA2 in *kat6a* mutants and heterozygotes.
